# Cortical spheroids show strain-dependent cell viability loss and neurite disruption following sustained compression injury

**DOI:** 10.1371/journal.pone.0295086

**Published:** 2024-08-19

**Authors:** Rafael D. González-Cruz, Yang Wan, Amina Burgess, Dominick Calvao, William Renken, Francesca Vecchio, Christian Franck, Haneesh Kesari, Diane Hoffman-Kim

**Affiliations:** 1 Department of Neuroscience, Brown University, Providence, RI, United States of America; 2 Carney Institute for Brain Science, Brown University, Providence, RI, United States of America; 3 School of Engineering, Brown University, Providence, RI, United States of America; 4 Institute for Biology, Engineering, and Medicine, Brown University Providence, RI, United States of America; 5 Center for Traumatic Brain Injury, University of Wisconsin-Madison, Madison, WI, United States of America; 6 Department of Mechanical Engineering, University of Wisconsin-Madison, Madison, WI, United States of America; Università degli Studi della Campania, ITALY

## Abstract

Sustained compressive injury (SCI) in the brain is observed in numerous injury and pathological scenarios, including tumors, ischemic stroke, and traumatic brain injury-related tissue swelling. Sustained compressive injury is characterized by tissue loading over time, and currently, there are few *in vitro* models suitable to study neural cell responses to strain-dependent sustained compressive injury. Here, we present an *in vitro* model of sustained compressive neural injury via centrifugation. Spheroids were made from neonatal rat cortical cells seeded at 4000 cells/spheroid and cultured for 14 days *in vitro*. A subset of spheroids was centrifuged at 104, 209, 313 or 419 rads/s for 2 minutes. Modeling the physical deformation of the spheroids via finite element analyses, we found that spheroids centrifuged at the aforementioned angular velocities experienced pressures of 10, 38, 84 and 149 kPa, respectively, and compressive (resp. tensile) strains of 10% (5%), 18% (9%), 27% (14%) and 35% (18%), respectively. Quantification of LIVE-DEAD assay and Hoechst 33342 nuclear staining showed that centrifuged spheroids subjected to pressures above 10 kPa exhibited significantly higher DNA damage than control spheroids at 2, 8, and 24 hours post-injury. Immunohistochemistry of β_3_-tubulin networks at 2, 8, and 24 hours post-centrifugation injury showed increasing degradation of microtubules over time with increasing strain. Our findings show that cellular injuries occur as a result of specific levels and timings of sustained tissue strains. This experimental SCI model provides a high throughput *in vitro* platform to examine cellular injury, to gain insights into brain injury that could be targeted with therapeutic strategies.

## Introduction

Sustained compression injury in the brain is a feature of many brain diseases and injuries [1, 2]. Increased intracranial pressure (ICP) exerted from expanding brain tumors, increased hematoma edema volume, and/or swollen injured tissue [3] can injure neighboring tissues and their cells by deforming them. These deformations can be compressive as well as tensile. While the pressures generated in sustained compression injury (SCI) deform adjacent brain tissues at generally lower strain magnitudes and rates than those observed in blunt and blast head trauma, they typically last longer, ranging anywhere from minutes to months [4]. Prolonged pressures can result in damage at the cellular level and, eventually, lead to altered electrophysiology, chronic neuroinflammation, and neuronal death. However, there is neither a clear understanding of the magnitude of the resulting deformations caused by these pressures nor of their durations. This knowledge gap represents a hindrance to understanding SCI progression and developing clinical strategies to prevent long-term brain damage caused by sustained compression.

Most compressive brain injury studies, either *in vivo* or *in vitro*, focus on replicating traumatic brain injury conditions by applying forces for very short durations at high speeds to mimic varying real-life injury scenarios. Such studies have allowed scientists to gain insight into the events following injuries, from blunt impact to blast-induced injury, as a function of strain and strain rate. For example, *in vivo* studies employing setups such as controlled cortical impact injuries on rats have allowed for monitoring the progression of neuronal disruption [5] and neuroinflammation [6, 7] as well as injury biomarker release in live animals subjected to TBI [8]. Meanwhile, *in vitro* experiments have allowed monitoring of live cell responses to compressive injury to determine injury thresholds based on strain magnitude and strain rate in both two- and three-dimensional culture platforms [9–13]. While these studies provide information into the progression of strain-dependent brain injury, they typically employ quick loading and unloading conditions, and do not represent SCI.

Previous studies specific to SCI have been conducted *in vivo* using animal models and *in vitro* using culture platforms. *In vivo* SCI models include using setups similar to controlled cortical impact, but delaying unloading times to minutes rather than milliseconds [14] and implantation of a hemispherical plastic bead inside rat somatosensory cortices to compress the brain statically [15]. *In vitro* SCI studies have typically applied pressurized air to 2D cultures of neural cell lines in pressurized culture chambers [16, 17]. These studies have begun to provide a time window into cellular events that occur after the exposure of the brain and neuronal cells to forces. However, there is a need for *in vitro* models that can achieve the combination of (1) controlling the pressures required to introduce strains; (2) monitoring the resulting effects over time; and (3) doing so in a robust 3D model that recapitulates key features of the brain.

In this study, we present a new in *vitro injury* model as a platform to study sustained compression injury and its progression within the first 24 hours post-injury. We used a three-dimensional *in vitro* cortical spheroid [18–20] which mimics multiple characteristics of the *in vivo* brain that are highly relevant to injury response. Generated from postnatal rat cortical cells by self-assembly, the cortical spheroid contains interconnected cell types, cell density, and tissue stiffness of the *in vivo* cortex. Spheroid neurons are electrically active, with mature synapses and neurite networks in the first two weeks of culture. Spheroid cells include neurons, astrocytes, oligodendrocytes, microglia, and endothelial cells, and produce extracellular matrix, myelin, as well as capillary-like networks. All these characteristics are reproducible, and one postnatal rat cortex yields hundreds of spheroids, making this model an excellent choice for high throughput testing.

Here, cortical spheroids were deformed by pressure generated via centrifugation. The pressures were modulated by varying the centrifugation speeds, measured with pressure gauges, and computed via finite element analysis (FEA). We demonstrated that sustained compression injury caused cellular death and neuronal network disruption in cortical spheroids within the first 24 hours following compression injury. This timeframe is of interest due to its relevance for potential clinical intervention, as injury biomarkers related to poor clinical outcomes appear in patient biofluids just hours after a TBI event [21, 22]. Mechanics modeling and FEA allowed for the estimation of strains associated with the centrifugation-induced pressures and the concomitant changes observed in cell viability and neurite network organization. These results suggest that this model can facilitate the study of the effects of sustained compression and its resulting strains on the brain at the tissue and cellular levels.

## Materials and methods

### Cortical dissection and spheroid culture

**Fig 1:**
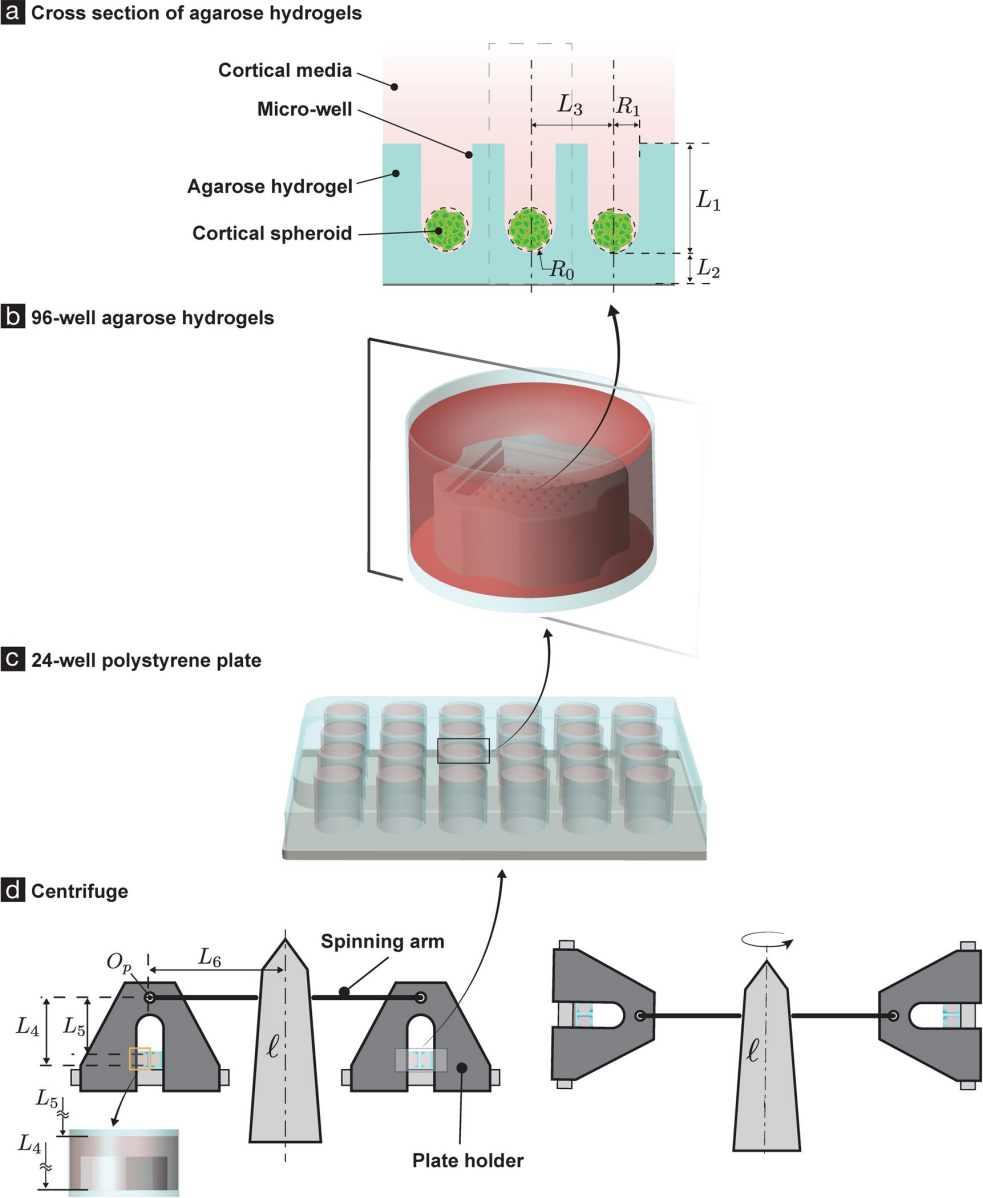
Experimental SCI setup and geometry of the spheroid centrifugation. DIV14 cortical spheroids were subjected to sustained compression injury via centrifugation. (a)–(d) show the geometry of the spheroids’ centrifugation. The spheroids’ radii (*R*_0_) varied from 75 to 85 microns. In our mechanics model (Fig 2) we take the spheroid’s radius to be 80 microns. Each spheroid lies in a microwell that is 800 microns deep (*L*_1_) and has a circular cross-section of radius 200 microns (*R*_1_), with a hemispherical base. The micro-well is part of an agarose hydrogel structure (shown in cyan in (a)). The thickness of the agarose material under a spheroid is 1.5 mm (*L*_2_). The agarose hydrogel contains 96 micro-wells in total, arranged in a square grid (b). The distance between adjacent micro-wells is *L*_3_, where *L*_3_ = 800 microns (a). The agarose hydrogel sits in one of the wells of a 24-well polystyrene plate (c). The polystyrene plate sits in a centrifuge cell culture plate holder (d), which is suspended from a centrifuge’s spinning arm. We refer to the point at which the centrifuge’s spinning arm attaches to the centrifuge cell culture plate holder as *O*_*p*_ (d). We measured the base of the agarose hydrogel to be at a distance of 66 mm from the point *O*_*P*_.(*L*_4_). The spheroid and the microwell were submerged in cortical fluid media. From the dimensions of the polystyrene well, the volume of the agarose hydrogel structure, and the amount of fluid media added to a polystyrene well, we estimate that the surface of the cortical fluid media is at a distance of 59.61 mm from *O*_*P*_. (*L*_5_). The length of the spinning arm is 112.69 mm (*L*_6_).

Cortical spheroids were assembled and cultured as described elsewhere [18], with minor modifications, from postnatal rat pups, while following university-approved IACUC protocols. Briefly, 4–6 P2 CD neonatal male and female pups (Charles River) were anesthetized by hypothermia, confirmed via paw pinching and then euthanized via decapitation with sharp scissors. Once euthanized, rat pup cortices were dissected and placed in cold Hibernate A with B27 Plus Supplement (Invitrogen). Then they were minced into small pieces and transferred to a 2 mg/ml papain solution, which was composed of lyophilized papain powder and Hibernate A without calcium chloride (BrainBits, LLC) and pre-warmed to 30°C. Cortical digestion in papain lasted for 30 minutes, with 5-minute agitation intervals. Single cortical cell suspensions were obtained by filtering and centrifuging the digested cell suspension at 150 RPM and seeded at 4000 cells/well in 96-well agarose hydrogels (see Fig 1A and 1B) to form cortical spheroids (see Fig 1A) via self-assembly. The agarose hydrogels were made by pouring a volume of 2% w/v molten agarose onto silicone molds (#24-96-Small, MicroTissues Inc), transferred to a 24-well polystyrene plate (see Fig 1C) (CELLTREAT), and equilibrated with complete cortical media for 48 hours at 37°C prior to cell seeding. The 2% w/v agarose solution was made by dissolving 2g of ultrapure agarose powder (Invitrogen) in 100 mL of 1X phosphate buffer saline, pH = 7.4 (Gibco) and heating the 2% agarose solution in a microwave for 1 minute to solubilize the agarose powder. Self-assembled spheroids were cultured for 14 days *in vitro* (DIV14) at 37°C in complete cortical media. Complete cortical media consisted of Neurobasal A+ (Invitrogen), 1x B27 Plus Supplement, 0.5 mM GlutaMAX, and 1x Pen/Strep (Invitrogen).

### Centrifugation

DIV14 cortical spheroids were subjected to sustained compression injury via centrifugation (Fig 1). The spheroids were suspended in media in micro-wells of an agarose hydrogel structure (Fig 1A and 1B). The agarose hydrogel sat in one of the wells of a 24-well polystyrene plate (Fig 1C). Media was added to the wells of the polystyrene plate, so that the spheroids and the agarose-hydrogels were completely submerged by it. The size of the spheroid, the depth and thickness of the micro-well, the height of the fluid media in each polystyrene well, as well as other important dimensions in the SCI experiment are described in Fig 1‘s caption. The polystyrene plate sat in a centrifuge cell culture plate holder (Fig 1D), which was suspended from a centrifuge’s spinning arm. The centrifuge was of tissue culture-grade (5810R Eppendorf). The spheroids were mechanically loaded (and as we shall show later, injured) by being spun by the centrifuge.

In each mechanical loading the angular velocity *ω* radians/seconds (rads/s) of the centrifuge was varied with time as:

$$\left\{\begin{array}{c} \omega_{max}\frac{\tau }{\tau_{1}},\;\tau \in \left[0,\tau_{1}\right),\\ \omega_{max},\;\tau \in \left[\tau_{1},\tau_{2}\right),\\ \omega_{max}\left(1-\frac{\tau -\tau_{2}}{\tau_{3}-\tau_{2}}\right),\;\tau \in \left[\tau_{2},\tau_{3}\right). \end{array}\right.$$

That is, the loading consisted of three stages: (St.1) an acceleration stage (from 0 seconds to *τ*_1_ seconds) in which the angular velocity linearly increased from zero to *ω*_*max*_ rads/s; (St.2) a constant angular velocity stage (from *τ*_1_ seconds to *τ*_2_ seconds), in which the angular velocity was kept constant at *ω*_*max*_ rads/s, and; finally, (St.3) a deceleration stage (from *τ*_2_ seconds to *τ*_3_ seconds), in which the angular velocity decreased linearly from *ω*_*max*_ rads/s to zero.

The spheroids were subjected to four different mechanical loadings: *ω_max_*,_1_ = 104 (*τ*_1_ = 5, *τ*_2_ = 120, and *τ*_3_ = 124), *ω_max_*,_2_ = 209 (*τ*_1_ = 10, *τ*_2_ = 120, and *τ*_3_ = 134), *ω_max_*_3_ = 313 (*τ*_1_ = 17, *τ*_2_ = 120, and *τ*_3_ = 137), and *ω_max_*,_4_ = 419 (*τ*_1_ = 25, *τ*_2_ = 120, and *τ*_3_ = 144). These four angular velocities were chosen so that the whole range of speeds that our centrifuge was capable of accurately applying were explored uniformly. Our centrifuge was advertised as being capable of a maximum angular velocity of 5000 RPM. However, we expected it to be capable of accurately applying speeds only up to 4000 RPM. Therefore, we choose the speeds of 0 RPM (control), 1000 RPM (104 rads/s), 2000 RPM (209 rads/s), 3000 RPM (313 rads/s), and 4000 (419 rads/s) RPM in our experiments.

### Mechanics model of the cortical spheroid, agarose hydrogel, and the cortical fluid media system

We constructed and solved a model that captures the mechanical interaction between the spheroid, the agarose hydrogel, and the cortical fluid media as they are spun by the centrifuge. The primary goal of the model was to estimate the strains and the pressures in the spheroid during the second stage of the loading (St.2). In our model we assume that in a frame that rotates with the centrifuge’s rotating arm all spatial mechanical fields are stationary with respect to time. The model was developed using the mechanics formalism of [23–25]. The complete details of our model, including the various simplifying assumptions in it, are detailed in Supplementary Information (S1 Appendix) and [26].

**Fig 2:**
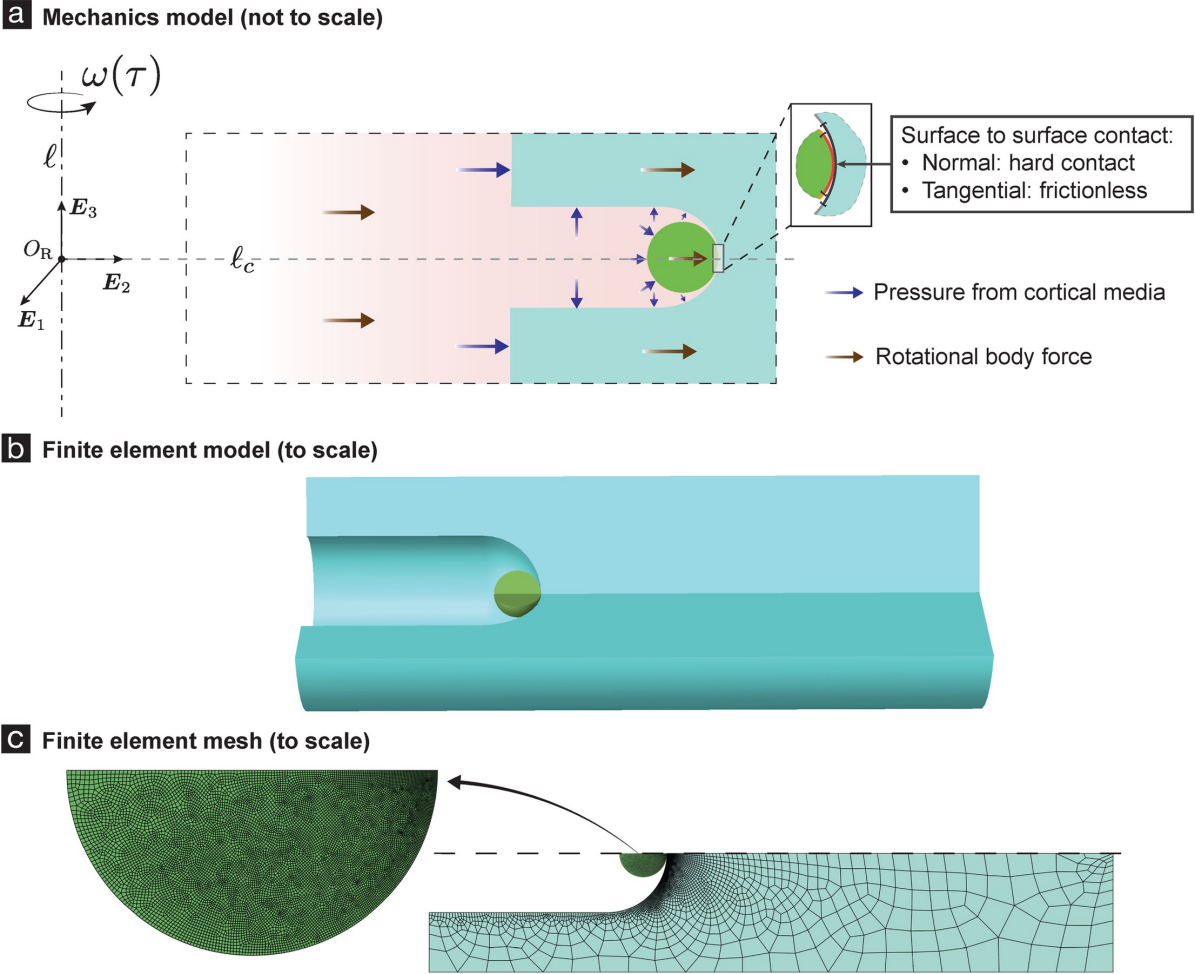
Mechanics model and finite element analysis of spheroid centrifugation (SCI experiments). **(a)** shows the geometry of our mechanics model of the SCI experiment. In our model we focus on a single spheroid. This is motivated by our assumption (a.1) that the stresses and strains in most of the spheroids are similar to each other (i.e., are of the same order of magnitude). We also assume (a.2) that the deformations and stresses in the spheroid and in the agarose hydrogel region in its vicinity are axi-symmetric. The spinning deforms and stresses the spheroid, and the agarose hydrogel, and creates additional pressures in the fluid media. We model this effect by introducing effective body forces (brown arrows in (a)) in each of them. We compute the pressure in the fluid media and use that to impose surface traction boundary conditions on the spheroid and the agarose hydrogel (purple arrows in (a)). We model the interaction between the spheroid and the agarose hydrogel using a non-adhesive, frictionless contact theory. The surface regions of the spheroid and the agarose hydrogel that eventually come into contact with each other are marked in red and dark-gray, respectively, in the inset of (a). **(b)** We use finite element (FE) techniques to solve the model in (a). The FE model contains a single spheroid and all the agarose hydrogel region that is at a distance of *L*_3_/2 microns from the central axis (*l*_*c*_) of the spheroid’s micro-well. We refer to this agarose hydrogel region as hydrogel cell (cyan in (b)). Recall that *L*_3_ is the distance between two adjacent agarose hydrogel microwells (see Fig 1 (A)). This feature of our FE model was motivated by our assumption (a.1). In the FE model we take all the mechanical fields to be axi-symmetric around the micro-well’s central axis. This feature is a consequence of our assumption (a.2). Also, as a consequence of this assumption the circumferential displacements in our FE model are naught. As a consequence of (a.1) and (a.2) we have that there are no radial displacements on the outer cylindrical surface of the hydrogel cell. We impose zero displacements at the base of the hydrogel cell. Other boundary conditions in our FE model are discussed in section *Centrifugation*. **(c)** shows a representative computational mesh of our FE model. The spheroid’s FE mesh typically contained around 11000 linear quadrilateral elements, and the hydrogel cell’s mesh typically contained around 3200 linear quadrilateral elements. After discretization and assembly, we solve around 29000 non-linear equations, using iterative numerical techniques.

Considering the spatial proximity of the cortical spheroids (see Fig 1B), while they are being centrifuged, we assume that all the spheroids experience the same order of magnitude of strains during the centrifugation. Consequently, we only model the deformation of a single cortical spheroid, along with the microwell containing it and the cortical media surrounding them. The geometry of the spheroid, the agarose hydrogel microwell, and the cortical fluid media in the reference configuration in our model is shown in Fig 2A. The important dimensions in that geometry are described in Fig 1, and their values are given in those figures’ captions.

We assume that the mechanics of the spheroid, the microwell composed of the agarose hydrogel, and the cortical fluid media can be well modeled using continuum mechanics theories. Consequently, we model the cortical spheroid and the agarose hydrogel microwell as homogenous solids, of densities 1240 mg/ml and 1640 mg/ml, respectively, and the cortical fluid media as a homogeneous fluid, of density 980 mg/ml. More specifically, we model the spheroid as a spherical ball composed of an incompressible neo-Hookean material of shear modulus 4/3 kPa; the micro-well as a structure composed of a compressible neo-Hookean material having shear modulus 107 kPa and bulk modulus 501 kPa; and the cortical media as an incompressible Newtonian fluid. It is unlikely that the spheroids have a purely elastic mechanical behavior, let alone the elastic behavior dictated by the incompressible neo-Hookean material model, as we assume here. However, since we do not have any direct and complete characterization of the spheroid’s mechanical behavior, we chose the incompressible neo-Hookean material model for the spheroid to simplify our analysis. We chose the value of 4/3 kPa for the spheroid’s shear modulus so that our model is consistent with previous studies’ data [27]. Our choices for the agarose-hydrogel-microwell and the cortical-fluid-media’s material models are also based on similar reasoning.

The spheroid, the micro-well, and the fluid media all experience effective body forces due to the spinning, see Fig 2A. We model their interactions with each other by applying to each of them suitable surface tractions and constraints on their deformations. In our model, we were able to explicitly compute the pressure field in the fluid media (see Section 4.3 in S1 Appendix and [26]). The interaction of the spheroid with the fluid media was captured by using that pressure field and constructing appropriate traction boundary conditions on the spheroid. The interaction of the microwell with the fluid media was captured similarly. The interaction between the spheroid and the microwell was modeled using non-adhesive frictionless contact boundary conditions. The other boundary conditions in our model are discussed in the caption of Fig 2.

We set up boundary value problems (BVPs) for the spheroid and the microwell based on the above discussed body forces, surface tractions, and contact boundary conditions. Their BVPs turn out to be coupled due to the contact boundary conditions. We solve for the strains and the pressures in the spheroid by solving the coupled boundary value problems simultaneously using nonlinear finite element techniques (see Fig 2B). See S1 Appendix and [26] for a more detailed description of our mechanics model. Representative pressures and strains in the spheroid, as predicted by our model, are shown in Fig 4.

### Pressure measurements

**Fig 3:**
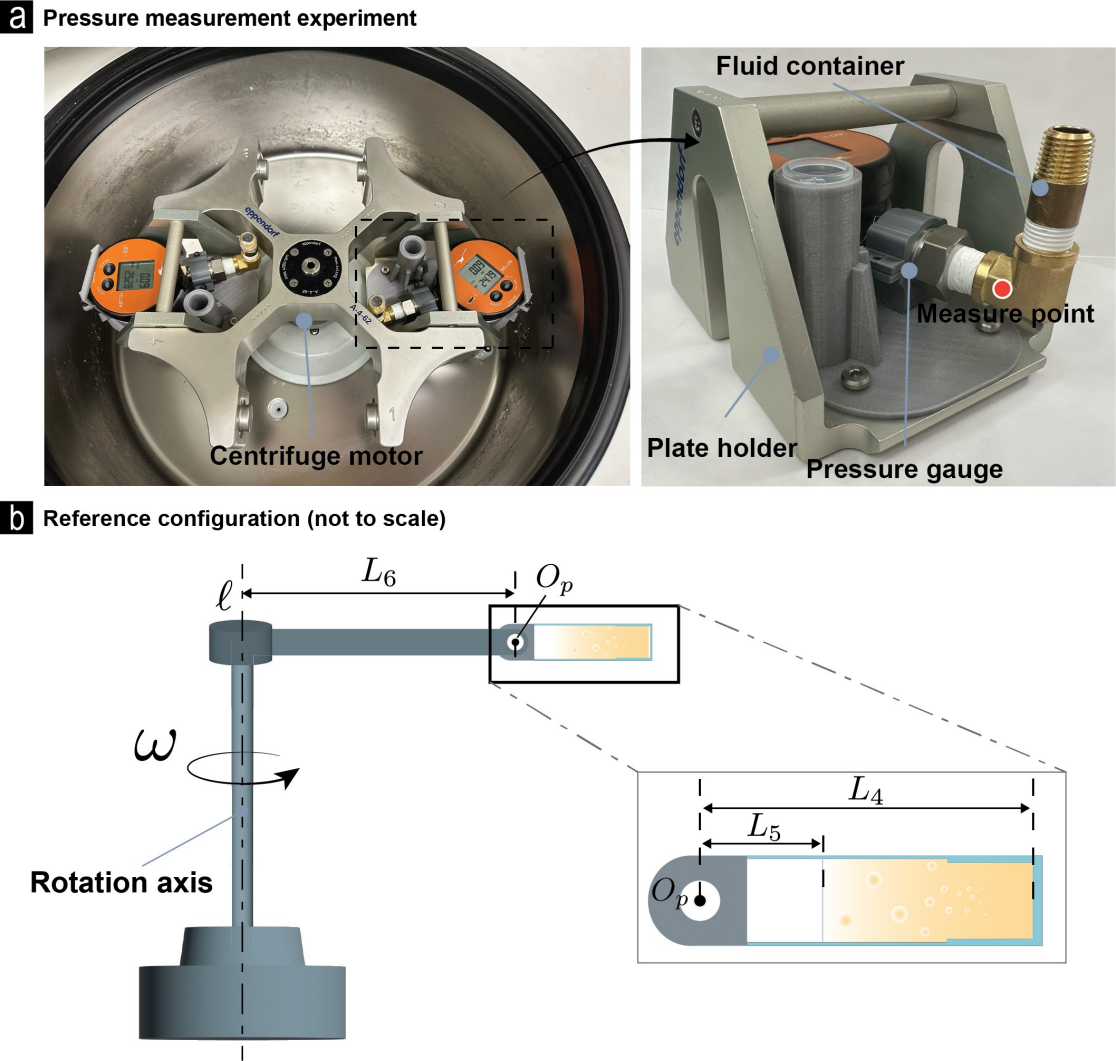
Set-up and geometry parameters of the pressure measurement experiments. The parameter *L*_7_ = *L*_5_ + *L*_6_, where *L*_5_ and *L*_6_ are shown marked in (b).

When the centrifuge is spinning at an angular velocity of *ω*, and the spheroids are submerged in a fluid media of density *ρ*_0_, we take the pressure, *p*, at a fluid particle X to be given by the formula

$$p=\frac{1}{2}\rho_{0}\omega^{2}\left({\left(X_{2}\right)}^{2}-{\left(L_{7}\right)}^{2}\right),$$ (1)

where *L*_7_ and *X*_2_ are length parameters (SI Fig (2) in S1 Appendix). The parameter *L*_7_ is the distance of the fluid media’s free surface from the centrifuge’s axis (also see Fig 3B). The parameter *X*_2_ denotes the distance of the fluid particle X from the centrifuge’s axis. Both of these distances are in the reference configuration shown in SI Fig (2) in S1 Appendix.

To measure pressures, scaled up versions of the SCI experiment were carried out (see Fig 3). In these experiments the micro-well and spheroid were replaced with a metal cylinder connected to a pressure sensor (LEO-1, Keller, US). The same centrifuge was used as in the SCI experiments. Water was the proxy for the fluid media. The speeds of the centrifuge were in the same range as the speeds used in the SCI experiments, from 1000–4000 RPM. Pressures were measured in a total of 75 experiments, in which the angular velocity and the height of the water column in the metal cylinder were varied.

### Sustained compression injury (SCI) experiments

To quantify cell death in centrifuged spheroids we incubated a subset of them for 0, 2, 8, and 24 hours after centrifugation and then stained them with 2 μM ethidium homodimer 1 (EthD1) in complete cortical media and 20 μg/mL Hoechst (Thermo Fisher Scientific) for 1 hour. Spheroids were rinsed twice with warm 1X PBS, treated with 1 mL of complete cortical media per well, and transferred from the agarose microwells into 35 mm-diameter Fluorodishes (World Precision Instruments) containing 1 mL of warm complete cortical media. To examine the effects of centrifugation-based injury in neuronal microtubule structures, we injured another subset of spheroids as described above and fixed overnight with 4% v/v paraformaldehyde and 8% w/v sucrose solution either at 0, 2, 8, and 24 hours post-injury. Following fixation, spheroids were transferred from the agarose wells into 1.5-mL Eppendorf centrifuge tubes, permeabilized and blocked using a 10% bovine serum albumin, 10% normal goat serum and 1% Triton 100-X for 2 hours. Then they were immunolabeled for β_3_-tubulin by treating them with mouse anti-β_3_-tubulin (BioLegend) primary antibodies at a 1:200 dilution overnight at room temperature. The following day, the spheroids were labeled Alexa Fluor 488-conjugated goat anti-mouse secondary antibodies (Jackson Laboratories) at a 1:500 dilution overnight and finally counterstained with 20 μg/mL DAPI solution. Immunolabeled spheroids were transferred to Fluorodishes containing PBS 1X.

### Confocal microscopy

Cortical spheroid images were acquired using an Olympus FV3000 laser scanning confocal microscope at a 30X magnification, oil immersion, 1.5-μm step size in z, and 50 slices per spheroid. Thirteen—twenty-nine spheroids per sample were imaged up to 75 microns depth. Images were acquired in Galvano mode. For living spheroid imaging, Fluorodishes containing complete cortical media were kept warm at 37°C by a plate adapter with a built-in thermocouple.

### Image processing and statistical analysis

Images were processed using Fiji v 2.11.0 software. Cell viability was determined by counting all EthD1- and Hoechst-labeled nuclei using the 3D Object Counter plug-in and using the following formula to calculate % cell viability:

$$\left(1-\frac{\#\;\text{dead}\;\text{cells}}{\text{Total}\;\#\;\text{of}\;\text{cells}}\right)\times 100\%$$

All results are reported as median ± interquartile range in the text. Data visualizations and statistical analysis were performed using GraphPad Prism 9 (GraphPad Software LLC). All data analysis was performed using one-way ANOVA with post-hoc Tukey-HSD tests.

## Results

### Sustained compression injury strains were determined by mechanics modeling and finite element analysis

**Fig 4:**
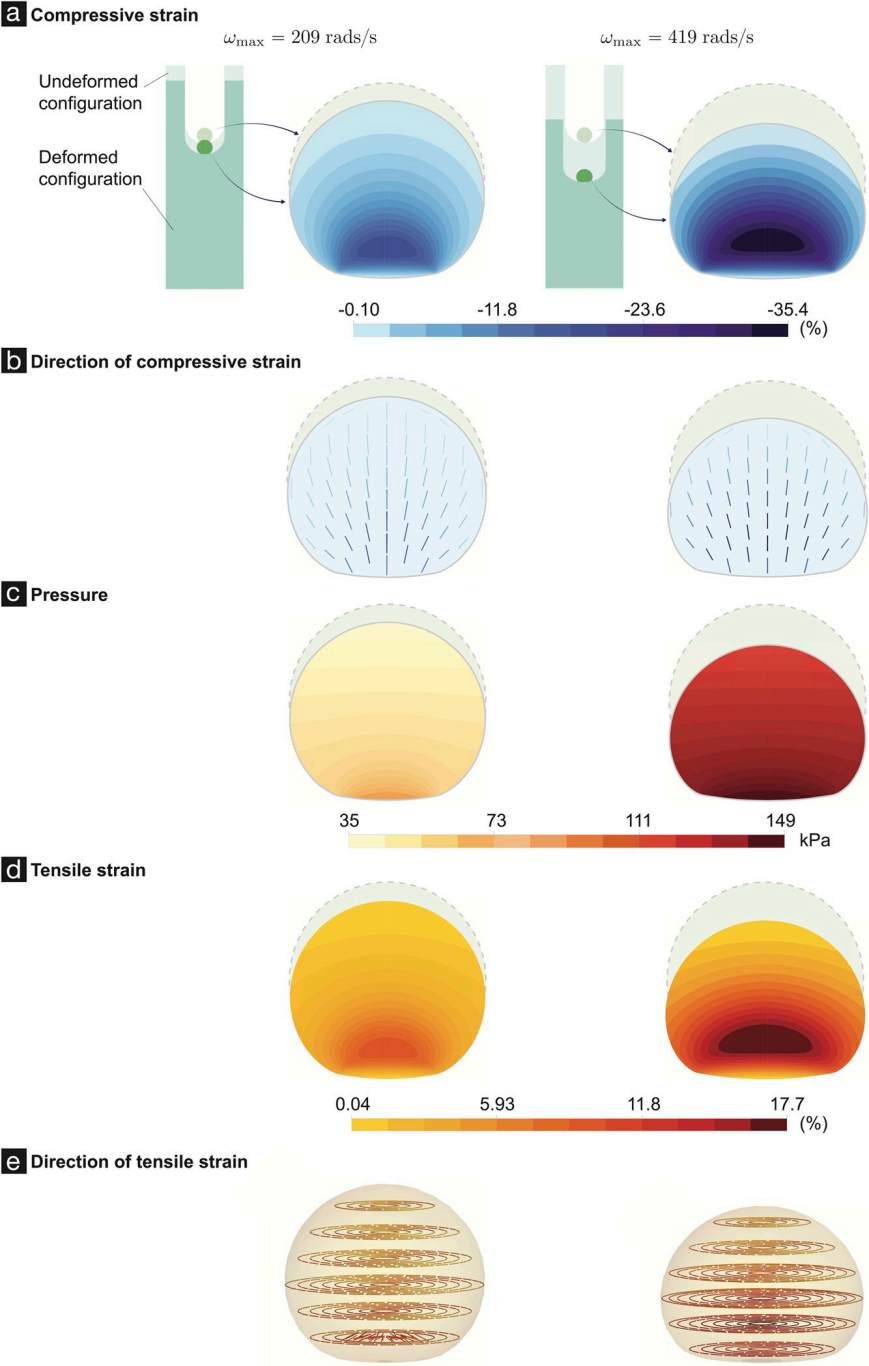
Deformations, strains, and pressures in a representative spheroid due to centrifugation at angular velocities of 209 rads/s (left column) and 419 rads/s (right column). (A) shows the compressive strain, i.e., the minimum eigenvalue of the logarithmic strain tensor. For details on the logarithmic strain tensor see [26]. (B) Each line segment shows a section of the fiber associated with the eigenvectors that correspond to the minimum eigenvalue of the logarithmic strain tensor. (C) shows the pressures in the spheroid. (D) shows the tensile strain, i.e., the maximum eigenvalue of the logarithmic strain tensor. (E) Each line segment shows a section of the fiber associated with the eigenvectors that correspond to the maximum eigenvalue.

Injury severity in sustained compression injury is determined by the strains and duration of compression events experienced by the cells in brain tissue, which can be difficult to determine when the compression-induced deformations cannot be visualized and measured experimentally. In this study, we cannot visualize the strain experienced by the cortical spheroids during centrifugation-induced sustained compression. To estimate said strains, we relied on mechanics modeling and finite element analysis, as detailed in Supplementary Information and [26]. We estimated that centrifugation speeds of 104 rads/s, 209 rads/s, 313 rads/s, and 419 rads/s generated (peak, over the spheroid) pressures of 10, 38, 84, and 149 kPa, respectively. These created peak compressive strain magnitudes of 10%, 18%, 27%, and 35%, respectively (Fig 4A and 4B), which correspond to the minimum eigenvalue of the logarithmic strain tensor. Interestingly, despite the pressures throughout the spheroids remaining positive, our calculations reveal that the spheroids also experienced tensile strains (e.g., see Fig 4D and 4E). The compressive strains predominantly occur in the direction of the centrifuge’s rotating arm. If the centrifuge’s rotating arm direction is taken to be the z-axis of a cylindrical co-ordinate system, then the tensile strains predominantly occur in the circumferential directions. We estimated peak tensile strain magnitudes of 5%, 9%, 14%, and 18%, respectively, in the four different angular velocity SCI experiments (these correspond to the maximum eigenvalue of the logarithmic strain tensor).

At the current stage it is unclear as to which particular strain measure(s) (compressive strain, tensile strain, etc.) are most pertinent to cellular death in SCI. However, since in our case the compressive and tensile strains scale with each other, we decided to use the compressive strain values when we want to refer to the magnitude of the deformations/strains in the spheroids.

### Pressure measurements showed high agreement with modeling estimates

To confirm the pressures used in the modeling estimates, we undertook pressure measurement experiments. The formula (1) can be written in the non-dimensional form

$$\frac{p}{\frac{1}{2}\rho_{0}\omega^{2}{\left(X_{2}\right)}^{2}}=\left(1-{\left(\frac{L_{7}}{X_{2}}\right)}^{2}\right).$$ (2)

As per the above formula the non-dimensional pressure variable $p/\left(\frac{1}{2}\rho_{0}\omega^{2}{\left(X_{2}\right)}^{2}\right)$ scales linearly with the non-dimensional (non-linear) measure of the fluid column height 1−(*L*_7_*/X*_2_)^2^ with a proportionality constant of unity.

In the pressure measurement experiments, the data points measured were of the form (*p*,*ω*,*i*), where *p* and *ω* are, respectively, the pressure and angular velocity. The symbol *i* denotes an integer that belongs to the range 1–4. It relates to *L*_7_ as

$$L_{7}=L_{7}^{\circ }+i\Delta h,$$ (3)

where $L_{7}^{\circ }$ and *h* are length parameters that remained constant over the experiments. The parameter *X*_2_ was also kept constant over the experiments. If the collected data were to be in perfect agreement with formula (2), then each of the data points

$$\left(\frac{p}{\frac{1}{2}\rho_{0}\omega^{2}{\left(X_{2}\right)}^{2}},1-{\left(\frac{L_{7}^{\circ }+i\Delta h}{X_{2}}\right)}^{2}\right)$$ (4)

would have to lie on a straight line of slope unity and passing through the origin.

**Fig 5:**
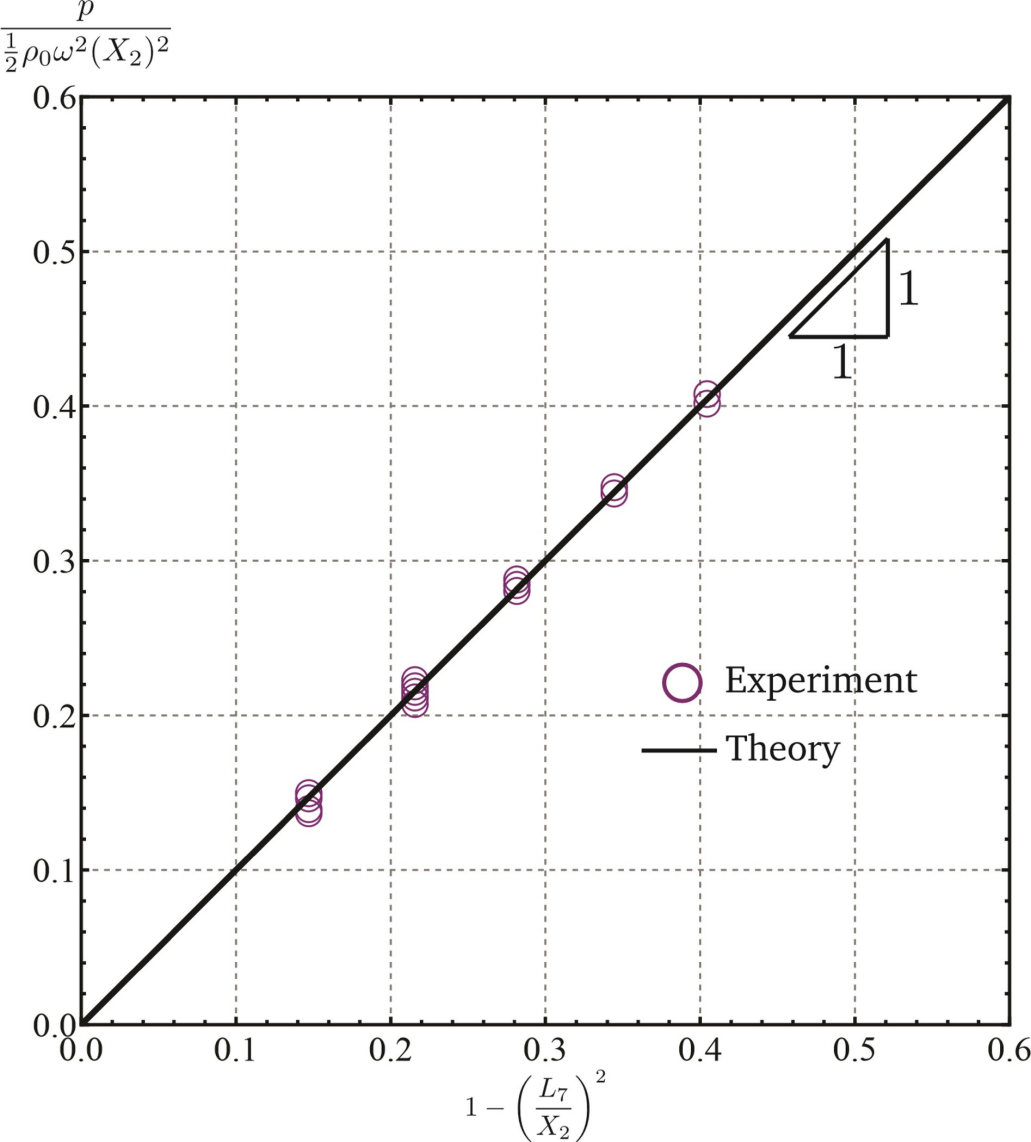
Results from the pressure measurement experiments. The measurements are in strong agreement with the formula (2).

Three of the data points (*p*,*ω*,*i*) were used to get a more accurate estimate for Δ*h*, $L_{7}^{\circ }$, and *X*_2_ in these experiments. Using these more accurate estimates each of the remaining 72 data points (*p*,*ω*,*i*) was converted into the form given in (4) (Fig 5). The data points lie on or are very close to the straight line passing through the origin and having a slope of unity (Fig 5). That is, the data from the pressure measurement experiments is in strong agreement with the formula (2).

### Cortical spheroids showed reduced cell viability following sustained compression

**Fig 6:**
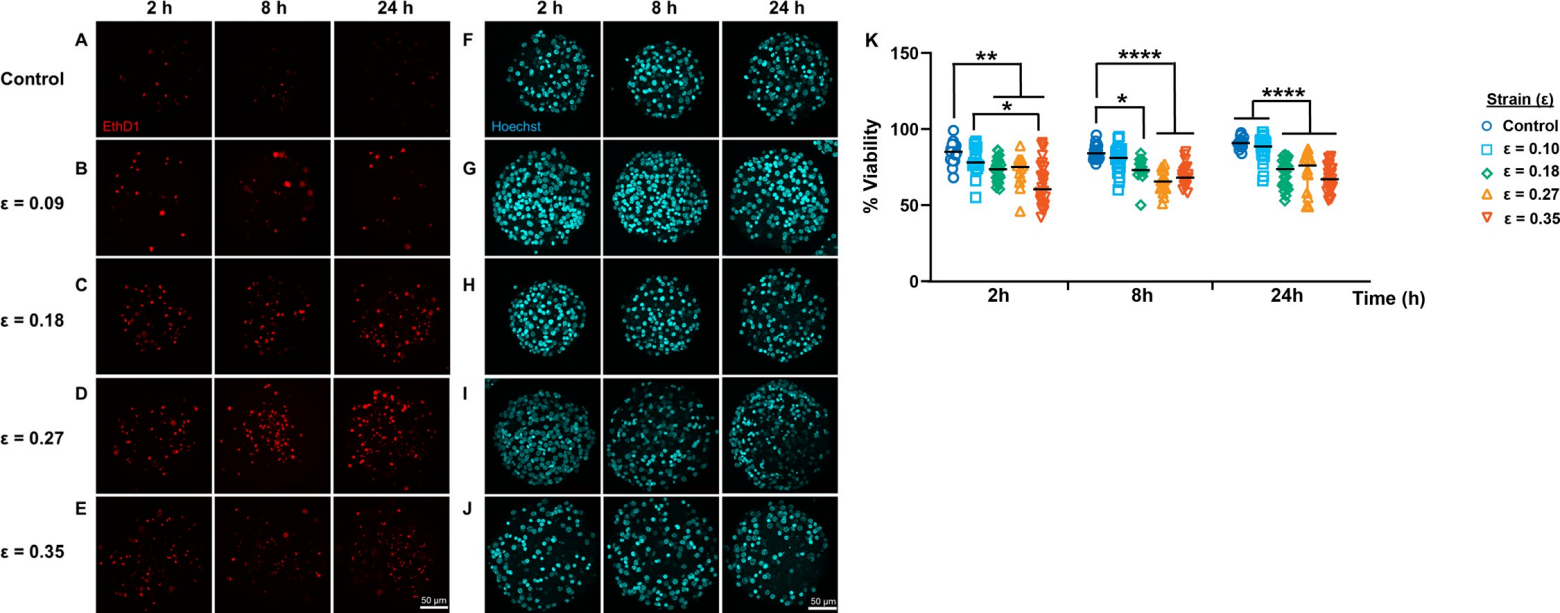
Cell viability loss following sustained compression in spheroids. Cortical spheroids were subjected to sustained compression injury via centrifugation at angular velocities of 104 rads/s (strain of 0.10), 209 rads/s (strain of 0.18), 313 rads/s (strain of 0.27), and 419 rads/s (strain of 0.35) for 2 minutes. Compressive strains (ε) resulting from these angular velocities were determined using mechanics modeling and FEA estimates. (A-J) Living cortical spheroids were labeled for dead cells and total cell nuclei using ethidium homodimer-1 (EthD1, shown in red) and Hoechst 33248 (shown in teal), and imaged 2, 8, and 24 hours post-injury using confocal laser microscopy. (K) Quantification of % cell viability in control and spheroids injured 2, 8, and 24 hours following sustained compression injury. Sample sizes for control samples, denoted with open blue circles, were n_2h_ = 25, n_8h_ = 23, n_24h_ = 18. Sample sizes for spheroids subjected to compressive strains of 0.10, denoted with open light blue squares, were n_2h_ = 18, n_8h_ = 25, n_24h_ = 28. Sample sizes for spheroids subjected to compressive strains of 0.18, denoted with open green diamonds, were n_2h_ = 27, n_8h_ = 13, n_24h_ = 27. Sample sizes for spheroids subjected to compressive strains of 0.27, denoted with open yellow triangles, were n_2h_ = 12, n_8h_ = 16, n_24h_ = 16. Sample sizes for spheroids subjected to strains of 0.35, denoted with open vermillion inverted triangles, were n_2h_ = 24, n_8h_ = 20, n_24h_ = 29. Data reported as median ± interquartile range. Asterisks denote statistical significance (* = *p* < 0.05, ** = *p* < 0.005, *** = *p* < 0.0005, **** = *p* < 0.0001).

Cellular death is a prominent event following mechanical insults such as sustained compression injury and is responsible for the onset of neuronal loss associated with cognitive loss [28]. In many brain injury studies, the injury has been deemed biphasic, with a primary injury of mechanical nature occurring immediately after trauma resulting from the pressures deforming the cells and a secondary injury caused by biochemical signaling triggered by injured cells hours following injury [29]. Therefore, we examined the results of sustained compression injury 2, 8, and 24 hours to capture this injury timeline. We found that centrifugation-enabled sustained compression reduced cell viability in cortical spheroids in a strain-dependent manner, with the highest reduction observed 2 hours post-injury (Fig 6). Recall that our calculations predict that spheroids compressed at 10, 38, 84 and 149 kPa experience compressive strains of10%, 18%, 27% and 35%, respectively. Spheroids compressed by 10% strain were statistically similar to controls throughout all time points (*p*_*2h*_ = 0.81, *p*_*8h*_ = 0.89, *p*_*24h*_ = 0.99). However, spheroids strained at higher strains exhibited significantly lower cell viabilities of 73±10%, 72±11%, and 60±24% following 2 hours post-injury when compared to time-matched control spheroids (85±8%, *p* < 0.0001). Similar viability results were observed at 8 and 24 hours when comparing injured spheroids compressed at 38, 84, and149 kPa to corresponding time-matched control spheroids. Spheroids compressed at 18%, 27%, and 35% strains exhibited significantly reduced cell viabilities of 73±8%, 65±8% and 68±10% following 8 hours post-injury and 74±10%, 76±14%, and 67±13% following 24 post-injury when compared to those of time-matched control spheroids (85±6%, 84±8% and 91±3%, *p* < 0.0001). Also, spheroids compressed at 35% strain had lower cell viability than those compressed at 18% strain 2 hours post-injury (p < 0.01) but exhibited similar cell viability reductions after 8- and 24-hours post-injury. These results suggest that most of the observed reduction in viability occurred early following sustained compression injury, where strain magnitude determined the severity of the observed cell viability loss.

### Cortical spheroids showed disrupted neurite networks following sustained compression

**Fig 7:**
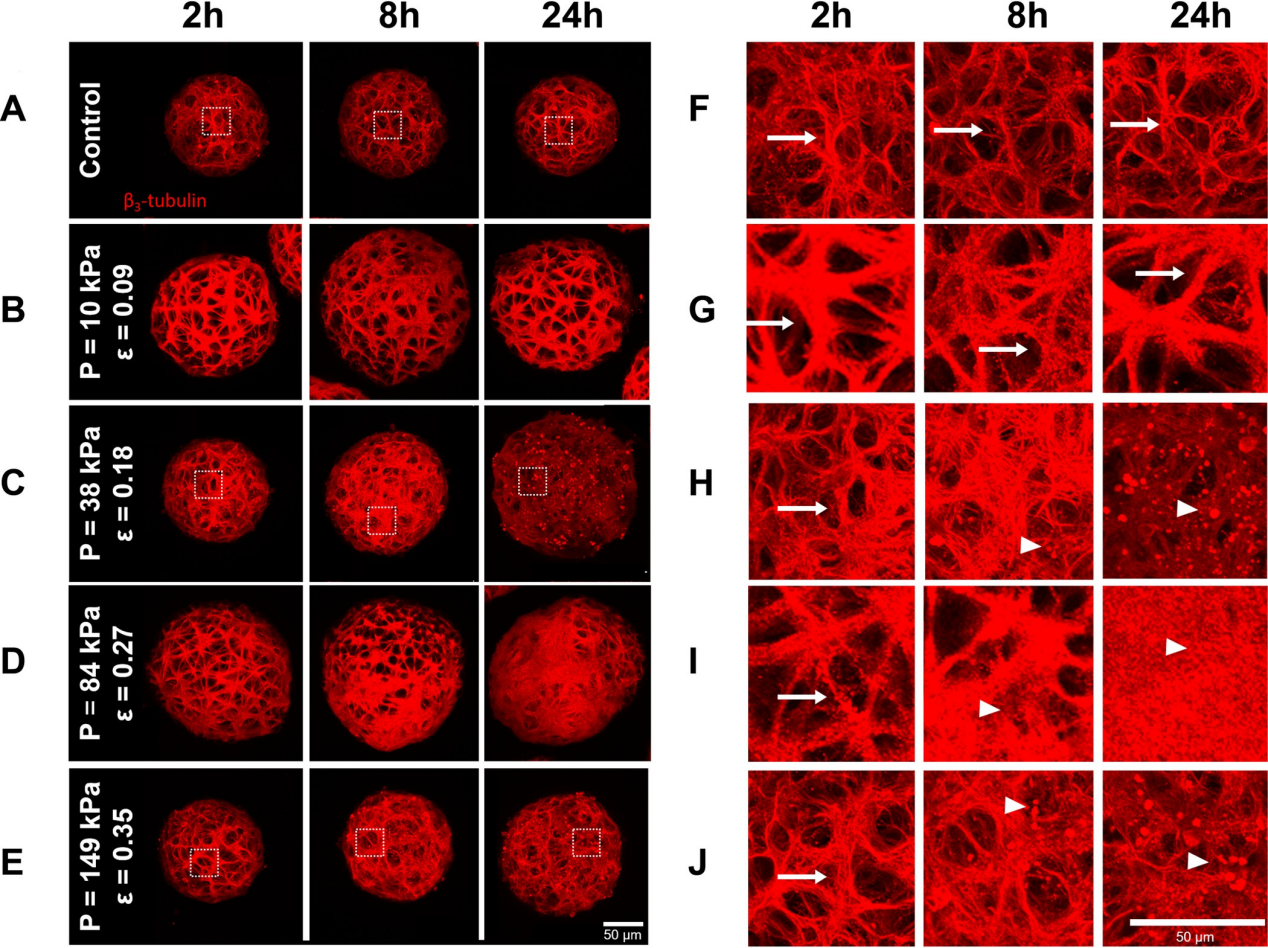
Neurite network disruption in cortical spheroids following sustained compression injury. Confocal z-projections of whole spheroids immunostained for β_3_-tubulin using mouse anti- β_3_-tubulin primary antibodies. Cortical spheroids were subjected to sustained compression injury via centrifugation at angular velocities of 104 rads/s (compressive strain of 0.10), 209 rads/s (compressive strain of 0.18), 313 rads/s (compressive strain of 0.27) and 419 rads/s (compressive strain of 0.35) for 2 minutes. Strains resulting from these angular velocities were determined using mechanics modeling and FEA estimates. Control (A, F) and injured spheroids (B, C, D, E, G, H, I, J) were fixed 2, 8, and 24 hours post-centrifugation. F-J show high magnification views of the corresponding boxed regions in A-E. Arrows represent intact microtubule networks while arrowheads represent puncta. Scale bars, 50 μm.

Secondary injury resulting from compressive brain trauma involves a delayed response characterized by biochemical signals that result in neuronal cell loss. Two hours following SCI, cortical microtissues subjected to sustained compression did not exhibit marked differences in neurite network organization and structure when compared qualitatively to control spheroids (Fig 7). However, 8 and 24 hours after injury, spheroids exposed to compressive strains greater than 0.10 exhibited a loss of the strands of their tubulin neurite networks. Neurite cytoskeletal structures became less defined with more punctate beta-3 tubulin staining, which is associated with microtubule degradation. Of note, this pattern of strain-dependent injury response to compressive strains above 0.10 paralleled that observed in our cell viability experiments.

## Discussion

Our aims for this study were to highlight a novel centrifugation-based, sustained compression injury modality subjecting cortical spheroids to sustained pressures at low speeds, and to characterize the strains caused by sustained compression using physics-based modeling and finite element analysis. We examined the effects of these strains on cortical neurospheroid cell viability and neurite network organization to observe if sustained pressures achieved via centrifugation would trigger injurious events at the cellular level. Using mechanics-based modeling and FE calculation, we found that strains resulting from sustained compression injury fell in the range of strains reported in mild and moderate brain injury *in vitro* and *in vivo* models [30] as well as from finite element studies [31–33]. These strains significantly reduced cell viability and disrupted neuronal cytoskeletal organization in cortical spheroids within the first 24 hours following injury. These results suggest that this injury modality can be used to study sustained compression-based injury while having knowledge of tissue strains. Being able to couple strain information with temporal information on changes in cellular viability and neurite network morphology could provide future insight into injury thresholds and therapeutic time windows following the onset of primary and secondary injury cellular events.

In *in vivo* rodent models, cortical compression has led to increased oxidative stress, astrocyte reactivity, and blood-brain barrier permeability [15], and neuronal hyperexcitability [14]. Other *in vitro* studies of sustained compression have observed increased lactate dehydrogenase in response to pressurized 2D cell cultures with neuronal and astrocyte cell lines [16, 17]. In the present study, we showed strain-dependent decreased cell viability and disrupted neurite network organization in 3D multicellular cortical spheroids within 24 hours post-injury. This sustained compression injury experimental modality compressed all spheroids in the same plane while suspended in liquid media. No natural or synthetic hydrogel was used to surround the spheroids, as can be required in some brain injury experiments. The spheroids contained all the key cell types of the brain, including glia; the cells and neurites were interconnected; they produced their own extracellular matrix; and the neurons were electrically active, with mature synapses [18, 20]. We have recently demonstrated the utility of these cortical spheroids for modeling disease and injury effects, as they can respond to oxygen deprivation and ischemia [34]. While the spheroid does not provide the layer-specific organization of the *in vivo* cortex, it is an efficient, and importantly, a high-throughput approach that presents many key *in vivo* characteristics. Ongoing studies are examining the responses of the spheroid’s other cell types to sustained compression injury to leverage the model’s complexity.

Using the centrifuge to study sustained compression injury has several advantages. The spheroids are all likely to experience the same order of magnitude of pressures because they are seeded and self-assembled in the same plane. There are no concerns for anisotropy and heterogeneity in the liquid media. The fluid’s homogeneity and incompressibility facilitate accurate quantification of the pressures experienced by the spheroids. Finally, the centrifuge modality of injury allows for precise control of the pressures by varying the angular velocity and the height of the liquid column.

Our approach also has current limitations. The most prominent is that our calculations for the spheroid strains are likely to be only a first order estimate, for the following reasons. We model the spheroid as a (a) homogeneous solid composed of an (b) incompressible (c) neo-Hookean elastic material. Regarding assumption (a): A solid can be assumed to be homogenous if the length-scale at which it first starts being significantly heterogeneous is small compared to the other length scales in the problem. The length scale at which the spheroid starts being significantly heterogeneous (see Fig 5) is not that much smaller than its overall size. Regarding (b): All materials are compressible at appropriate pressures. Finally, regarding (c): The spheroid’s mechanical behavior is likely to have viscous, plastic, and poro-elastic components in addition to an elastic component. Hence, there are reasons to expect that our assumptions (a)–(c) do not hold perfectly in the experiments, and consequently that our calculations for the strains only provide first order estimates for them. Despite its limitations, our approach is very valuable, because of our approach’s several advantages described above. Further, we note that the estimates for the strains in our approach are only expected to improve in future SCI experiments, as the mechanical characterization of the spheroids becomes optimized.

Finally, we assessed the acute cellular response of the injury to validate our injury model given how many cellular events take place following primary injury just hours after TBI that are correlated to poor prognosis in both TBI patients [21, 22, 35–38] and animal models [39–41]. In human patients, the detection of injury biomarkers in blood, including astrocyte-specific glial fibrillary acidic protein (GFAP) and neuron-specific ubiquitin C-terminal hydrolase 1 (UCH-L1) and neurofilament light chain (Nf-L), has been found within the first 2 hours post-injury and their concentrations are linked to clinical outcomes. Given how early these intracellular proteins are detected, all these studies strongly indicate that an abrupt release of intracellular content from injured cells occurs within the first 24 hours post-injury. These findings guided our experimental plan to look at changes in viability and neuronal microtubule networks early post-injury to confirm cortical cell death in living spheroids and neuronal-specific cytoskeletal damage hours post-injury injury as a result of strain magnitude. We were able to do this because of our ability to visualize these dynamic injurious events in spheroids at specific time points early post-injury via confocal microscopy, which is experimentally complicated in animal models and virtually impossible in humans outside of postmortem tissue analysis. While examining injury responses beyond the acute stage of injury (> 24 hours) are not part of the current study, it would be of great interest to examine them in future studies using the novel injury modality presented here.

## Conclusion

In this work we have shown that for sustained compressive injury, the slow loading times involved during centrifugation resulted in low strain rates but still caused significant cellular damage. In contrast to the present work, other studies of brain injury often apply pressures for very short durations at high speeds, and strains and especially strain rates predominantly affect the injury severity (9,10). Our results suggest that in SCI, the deformation, or strain magnitude, is a predominant factor correlating with SCI severity.

The centrifuge modality of injury that we present in this paper is novel, and it can be expanded into a versatile platform for *in vitro* micro-tissue injury studies. It has high throughput, based on the hundreds of spheroids that can be simultaneously loaded. It lends itself to being easily modified so that other more sophisticated pressure time histories can be applied. Additionally, it can be used to study the effect of higher strain rates using more rapidly changing pressures. Finally, we believe that the ease of operation and accessibility of a lab-grade centrifuge, as well as the direct and simple way in which the pressures can be controlled during centrifugation, will make this injury modality relevant to future *in vitro* neurotrauma studies.

## Supporting information

S1 Appendix Solid mechanics-based modeling of the cortical spheroids’ deformation during centrifugation-induced compression injury. Fig 1: An illustration of the various mathematical objects that we use in our mechanics model of spheroid centrifugation. Fig 2: An illustration of the cortical spheroid (green), cortical fluid media (pink), and the agarose hydrogel microwell surfaces (cyan) in the deformed (a) and reference (b) configurations.
(PDF)
