# Supplementary material for: Cortical spheroids show strain-dependent cell viability loss and neurite disruption following sustained compression injury
Source: PLoS One. 2024 Aug 19;19(8):e0295086. doi: 10.1371/journal.pone.0295086 (PMC11332998; doi:10.1371/journal.pone.0295086)
Supplement: S1 Appendix — Fig 1: An illustration of the various mathematical objects that we use in our mechanics model of spheroid centrifugation. Fig 2: An illustration of the cortical spheroid (green), cortical fluid media (pink), and the agarose hydrogel microwell surfaces (cyan) in the deformed (a) and reference (b) configurations. (PDF) [file pone.0295086.s001.pdf]

Supporting Information–Sustained compression injury in cortical spheroids

1 Introduction

We constructed and solved a model that captures the mechanical interaction between the spheroid, the agarose hydrogel, and the cortical fluid media as they are spun by the centrifuge. The primary goal of the model was to estimate the strains and the pressures in the spheroid during the second stage of the loading.

The primary assumptions in our model are described in §2. The mathematical preliminaries necessary for detailing our model are described in §3. We develop the model in §4, and summarize it in §5.

2 Primary assumptions and modeling decisions, and their underlying rationale

In this section we list some of the primary assumptions and modeling decisions that we made for estimating the strains in the spheroid.

(a.1) Considering the spatial proximity of the cortical spheroids (see Fig. 1 (b) of the main manuscript), while they are being centrifuged, we assume that all the spheroids experience the same order of magnitude of strains during the centrifugation. **(d.1)** Consequently, we only model the deformation of a single cortical spheroid in this study.

(d.2) For modeling the deformation of a single spheroid, we also model the motion and deformation of the agarose hydrogel containing that spheroid, and the cortical fluid media surrounding the spheroid.

(a.2) We assume that the deformations and stresses in the spheroid and in the agarose hydrogel region in its vicinity are axi-symmetric.

(a.3) We assume that the mechanics of the spheroid, the agarose hydrogel, and the cortical fluid media can be well modeled using continuum theories [1]. **(d.3)** Consequently, we model the cortical spheroid and the agarose hydrogel as homogenous solids, and the cortical media as a homogeneous fluid. Even more specifically, **(d.3.i)** we model the spheroid as a spherical ball composed of an incompressible neo-Hookean material, **(d.3.ii)** the agarose hydrogel as a structure composed of a compressible neo-Hookean material, and the **(d.3.iii)** the cortical fluid media as an incompressible Newtonian fluid. The constitutive equation for the cortical fluid media is given in §4.3 and for the spheroid and agarose hydrogel are given in §4.4. Additionally, **(d.3.iv)** we model the interaction between the spheroid and agarose hydrogel as non-adhesive frictionless contact. We refer to the three mechanical entities *(i)* the cortical spheroid, *(ii)* the agarose hydrogel, and *(iii)* the cortical media collectively as the continua.

As we state in the main manuscript, each loading consists of three stages: **(St.1)** the first in which the centrifuge's angular velocity is linearly increased from naught to $\omega_{\max}$ over a period of τ_1 seconds; **(St.2)** the second in which the angular velocity is held fixed at $\omega_{\max}$ for a period of $\tau_2 - \tau_1$ seconds; and finally the third **(St.3)** in which the angular velocity is brought back to naught from $\omega_{\max}$ linearly over a

period of $\tau_3 - \tau_2$ seconds. As we shall show, in our model the angular velocities create effective body forces and surface tractions on the hydrogel and the spheroid (see §4.2.1 and §4.5). The magnitude of the forces and the tractions are proportional to the square of the magnitude of the angular velocity. Since the angular velocities during the first and the third stages are on the average smaller than the angular velocity during the middle stage by a factor of two, **(a.4)** we assume that the strains during the second stage are more important than the strains during the first and the third stages of the loading.

(d.4) On account of assumption *a.4* and for simplyfying the model we only estimate the strains during the second stage of the loading.

The accelerations of the material particles in the continua due to the centrifugation are greater than the acceleration due to gravity by a factor of about 800. **(d.5)** Therefore, we ignore acceleration due to gravity in our model.

(a.5) We assume that during the majority of the second stage of the loading in a frame that rotates with the centrifuge's arm, all mechanical fields remain stationary w.r.t. time.

(d.6) On account of assumption *a.3* and *a.5* it follows that the motion of the spheroid, agarose hydrogel, and the media can be described as

$$\boldsymbol{x}_{\boldsymbol{\tau}}[\boldsymbol{X}] = \boldsymbol{Q}_{\boldsymbol{\tau}} \boldsymbol{I}_{\mathbb{E}_{\mathrm{R}} \to \mathbb{E}} \left(\boldsymbol{X} + \boldsymbol{U}^{\star} \left[\boldsymbol{X} \right] \right). \tag{2.1}$$

Here $\boldsymbol{X}$ is the position vector of a continuum material particle $\mathscr{X}$ in a reference configuration. (The particle can belong to the spheroid, the agarose hydrogel, or the media.) We will henceforth be referring to the material particle $\mathscr{X}$ by its reference position vector, $\boldsymbol{X}$. We call $\boldsymbol{x}_{\boldsymbol{\tau}}[\cdot]$ the deformation map and $\boldsymbol{x}_{\boldsymbol{\tau}}[\boldsymbol{X}]$ the material particle $\boldsymbol{X}$'s current position vector at the time instance $\boldsymbol{\tau}$. We call $\boldsymbol{U}^{\star}[\boldsymbol{X}]$ the intermediate displacement of the material particle $\boldsymbol{X}$. In general the intermediate displacements in addition to $\boldsymbol{X}$ also depend on the time instance $\boldsymbol{\tau}$. In our model the intermediate displacements only depending on $\boldsymbol{X}$ is a consequence of our assumption *a.5*. We define the symbols $\boldsymbol{Q}_{\boldsymbol{\tau}}$, and $\boldsymbol{I}_{\mathbb{E}_{\mathrm{R}} \to \mathbb{E}}$ appearing in (2.1) in §4.1.2. For a mathematically complete and rigorous formulation of (2.1) see [2].

The material field $\boldsymbol{U}^{\star}[\cdot]$ is an unknown *a priori*. The strains and the stresses in the continua depend on the values of its gradient, $\{\boldsymbol{\nabla}_{\boldsymbol{X}}[\boldsymbol{U}^{\star}]\}[\cdot]$. In §4 we derive the equations whose solution will yield $\boldsymbol{U}^{\star}[\cdot]$, and hence the strains and the stresses.

3 Mathematical preliminaries

In this section we present the preliminary mechanics and mathematical notions that are needed for the development of our spheroid centrifugation model. Some of these notions can also be found in [3, §2.1] and [4, §2.1] .

3.1 Abstract vector spaces in our model

Let $\mathbb{E}_{\mathrm{R}}$ be an oriented Euclidean vector space, i.e., an oriented finite dimensional, real, inner product space, and let the affine point space $\mathscr{E}_{\mathrm{R}}$ have $\mathbb{E}_{\mathrm{R}}$ as its associated vector translation space. We refer to $\mathbb{E}_{\mathrm{R}}$ and $\mathscr{E}_{\mathrm{R}}$ as the reference Euclidean vector and point space, respectively. Let $\mathbb{E}$ and $\mathscr{E}$ be another pair of Euclidean vector and affine point space, respectively. Our continua (which can either be the spheroid, the agarose hydrogel, or the fluid media) execute their motion in $\mathscr{E}$. For that reason, we refer to $\mathbb{E}$ and $\mathscr{E}$ as the physical Euclidean vector space and point space, respectively. We model each of our continuum bodies, spheroid, agarose hydrogel, and the media using the topological spaces $\mathscr{B}^{\mathrm{sprd}}$, $\mathscr{B}^{\mathrm{gel}}$, and $\mathscr{B}^{\mathrm{fluid}}$, respectively (see Fig. 1 (a)).

We call a select continuous, injective map from $\mathscr{B}$ (which can be $\mathscr{B}^{\mathrm{fluid}}$, $\mathscr{B}^{\mathrm{sprd}}$, or $\mathscr{B}^{\mathrm{gel}}$) into $\mathbb{E}_{\mathrm{R}}$ the reference configuration and denoted it as $\boldsymbol{\kappa}_{\mathrm{R}}$. The elements of $\mathscr{B}$ are called material particles.

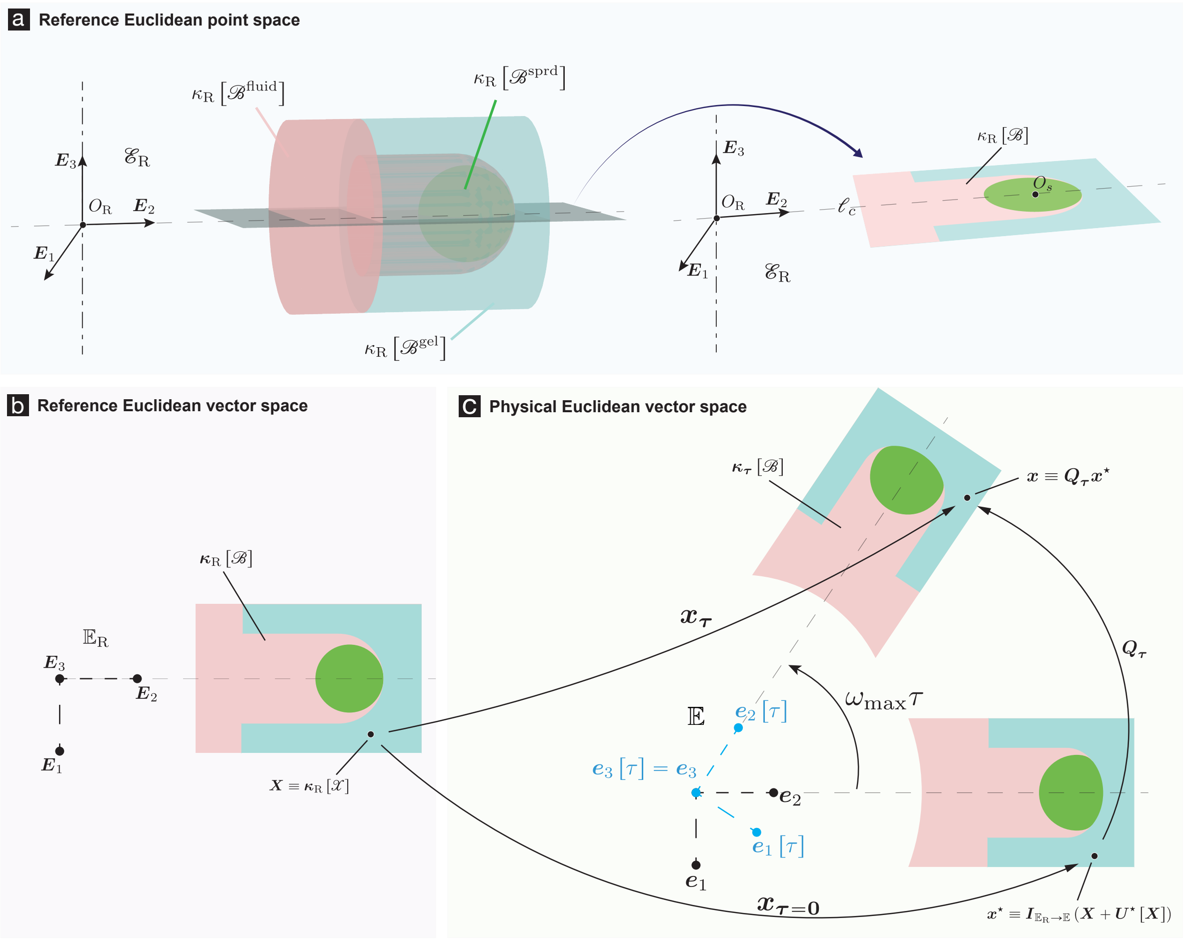

Figure 1 : An illustration of the various mathematical objects that we use in our mechanics model of spheroid centrifugation. All objects are defined in §3 and §4.1.

We call $\boldsymbol{X} \equiv \boldsymbol{\kappa}_{\rm R}\left[\mathscr{X}\right]$ the particle $\mathscr{X}$'s reference position vector and $\boldsymbol{\kappa}_{\rm R}\left[\mathscr{B}\right]$ the reference body. Taking some arbitrary point $O_{\rm R} \in \mathscr{E}_{\rm R}$ to be $\mathscr{E}_{\rm R}$'s origin, to $\boldsymbol{\kappa}_{\rm R}$ we associate the map $\kappa_{\rm R} : \mathscr{B} \to \mathscr{E}_{\rm R}$ such that $O_{\rm R} + \boldsymbol{\kappa}_{\rm R}\left[\mathscr{X}\right] = \kappa_{\rm R}\left[\mathscr{X}\right]$. We call $X \equiv \kappa_{\rm R}\left[\mathscr{X}\right]$ the particle $\mathscr{X}$'s reference point.

We model time as a one-dimensional normed vector space $\mathbb{T}$ and denote a typical element in it as $\boldsymbol{\tau} = \tau \boldsymbol{s}$, where $\tau \in \mathbb{R}$ and $\boldsymbol{s}$ is a fixed vector which has units of seconds.

3.2 Cartesian basis vectors

The sets $(\boldsymbol{E}_i)_{i\in\mathscr{I}}$ and $(\boldsymbol{e}_i)_{i\in\mathscr{I}}$, where $\mathscr{I} := (1, 2, 3)$, are orthonormal sets of basis vectors for $\mathbb{E}_{\rm R}$ and $\mathbb{E}$, respectively. By orthonormal we mean that the inner product between $\boldsymbol{E}_i$ and $\boldsymbol{E}_j$, or $\boldsymbol{e}_i$ and $\boldsymbol{e}_j$, where $i, j \in \mathscr{I}$, equals δ_{ij}, the Kronecker delta symbol, which equals unity iff $i = j$ and zero otherwise. In our problem, we take $\boldsymbol{E}_i$ and $\boldsymbol{e}_i$, $i \in \mathscr{I}$, to have the units of meters. The Cartesian co-ordinates of X which we denote as $\check{\mathsf{X}}\left[X\right] = \left(\check{\mathsf{X}}_i\left[X\right]\right)_{i\in\mathscr{I}}$, are components of $\boldsymbol{X}$ w.r.t. $\boldsymbol{E}_i$, that is $\check{\mathsf{X}}_i\left[X\right] = X_i$, where $X_i := \boldsymbol{X} \cdot \boldsymbol{E}_i$. For simplicity, $\mathsf{X} \equiv (X_1, X_2, X_3)$.

We denote the space of all $m \times n$ real nested ordered sets, where $m, n \in \mathbb{N}$, as $\mathscr{M}_{m\times n}(\mathbb{R})$. Thus $\check{\mathsf{X}}\left[X\right] \in \mathscr{M}_{3\times 1}(\mathbb{R})$. We call the map $\mathscr{E}_{\rm R} \ni X \mapsto \check{\mathsf{X}}\left[X\right] \in \mathscr{M}_{3\times 1}(\mathbb{R})$ the Cartesian co-ordinate map. Let $(\mathsf{x}_i)_{i\in\mathscr{I}}$ be orthonormal sets of basis vectors for $\mathscr{M}_{3\times 1}(\mathbb{R})$, or $\mathbb{R}^3$, where $\mathsf{x}_1 := (1, 0, 0)$, $\mathsf{x}_2 := (0, 1, 0)$, and $\mathsf{x}_3 := (0, 0, 1)$. When we refer to $\boldsymbol{X} \in \mathbb{E}_{\rm R}$, $\mathsf{X} \in \mathscr{M}_{3\times 1}(\mathbb{R})$, or $X \in \mathscr{E}_{\rm R}$ as a material particle we in fact mean the material particle $\mathscr{X} \in \mathscr{B}$.

3.3 Co-rotational basis vectors for $\mathbb{E}$

Let

$$\left(Q_{ij}\left[\tau\right]\right)_{i,j\in\mathscr{I}} = \begin{pmatrix} \cos\left[\omega_{\max}\tau\right] & -\sin\left[\omega_{\max}\tau\right] & 0 \\ \sin\left[\omega_{\max}\tau\right] & \cos\left[\omega_{\max}\tau\right] & 0 \\ 0 & 0 & 1 \end{pmatrix} =: \mathsf{Q}\left[\tau\right]. \tag{3.1}$$

The matrix $\mathsf{Q}[\tau]$ belongs to the specical orthonormal group $SO(3) \subset \mathscr{M}_{3\times 3}(\mathbb{R})$, and therefore satisfies the equations

$$\mathsf{Q}^{\mathsf{T}}\left[\tau\right]\,\mathsf{Q}\left[\tau\right] = \mathsf{I}_{3\times 3}, \tag{3.2a}$$

and

$$\mathsf{Q}\left[\tau\right]\,\mathsf{Q}^{\mathsf{T}}\left[\tau\right] = \mathsf{I}_{3\times 3}, \tag{3.2b}$$

where $\mathsf{Q}^{\mathsf{T}}[\tau]$ is the transpose of $\mathsf{Q}[\tau]$, i.e., $\mathsf{Q}^{\mathsf{T}}[\tau] = (\mathsf{Q}[\tau])^{\mathsf{T}}$ and $\mathsf{I}_{3\times 3} = (\delta_{ij})_{i,j\in\mathscr{I}} \in \mathscr{M}_{3\times 3}(\mathbb{R})$.

Using $\mathsf{Q}[\tau]$ we define the co-rotational set of basis vectors for $\mathbb{E}$, $(\boldsymbol{e}_i[\tau])_{i\in\mathscr{I}}$, as

$$\boldsymbol{e}_i\left[\tau\right] = Q_{ji}\left[\tau\right]\boldsymbol{e}_j. \tag{3.3}$$

Note that $(\boldsymbol{e}_i[\tau])_{i\in\mathscr{I}}$ change with time (see Fig. 1 (c)). However, at each time instance τ they form an orthonormal set of vectors and provide a basis for $\mathbb{E}$.

3.4 Co-rotational Cartesian co-ordinates

Given $\boldsymbol{x} \in \mathbb{E}$, let

$$\breve{x}_i\left[\boldsymbol{x},\tau\right] := \boldsymbol{x}\cdot\boldsymbol{e}_i\left[\tau\right], \tag{3.4}$$

where $(\boldsymbol{e}_i[\tau])_{i\in\mathscr{I}}$ is defined via (3.3). We call $(\breve{x}_i[\boldsymbol{x},\tau])_{i\in\mathscr{I}} =: \breve{\mathsf{x}}[\boldsymbol{x},\tau]$ the co-rotational Cartesian co-ordinates of $\boldsymbol{x}$ at the time instance τ.

3.5 Linear maps between vector spaces

Say $\mathbb{W}$ and $\mathbb{U}$ are two arbitrary, oriented Euclidean vector spaces; for instance, they can be $\mathbb{E}_{\mathrm{R}}$ and $\mathbb{E}$. We denote the space of all linear maps (transformations/operators) from $\mathbb{W}$ to $\mathbb{U}$ as $\mathscr{L}(\mathbb{W},\mathbb{U})$. We denote the norm of a vector $\boldsymbol{w}_1$ in $\mathbb{W}$ that is induced by $\mathbb{W}$'s inner product, i.e., $(\boldsymbol{w}_1\cdot\boldsymbol{w}_1)^{1/2}$, as $\|\boldsymbol{w}_1\|$. For $\boldsymbol{u}_1 \in \mathbb{U}$, the expression $\boldsymbol{u}_1 \otimes \boldsymbol{w}_1$ denotes the linear map from $\mathbb{W}$ to $\mathbb{U}$ defined as

$$\left(\boldsymbol{u}_1\otimes\boldsymbol{w}_1\right)\boldsymbol{w}_2 = \boldsymbol{u}_1\left(\boldsymbol{w}_1\cdot\boldsymbol{w}_2\right), \tag{3.5}$$

where $\boldsymbol{w}_2 \in \mathbb{W}$. If the sets $(\boldsymbol{u}_i)_{i\in\mathscr{I}}$ and $(\boldsymbol{w}_i)_{i\in\mathscr{I}}$ provide bases for $\mathbb{U}$ and $\mathbb{W}$, respectively, then it can be shown that $\left((\boldsymbol{u}_i\otimes\boldsymbol{w}_j)_{j\in\mathscr{I}}\right)_{i\in\mathscr{I}}$, which we will henceforth abbreviate as $(\boldsymbol{u}_i\otimes\boldsymbol{w}_j)_{i,j\in\mathscr{I}}$, provides a basis for $\mathscr{L}(\mathbb{W},\mathbb{U})$. The number T_{ij}, where $i,j \in \mathscr{I}$, is called the component of $\boldsymbol{T} \in \mathscr{L}(\mathbb{W},\mathbb{U})$ w.r.t. $\boldsymbol{u}_i\otimes\boldsymbol{w}_j$ iff $T_{ij} = \boldsymbol{u}_i\cdot(\boldsymbol{T}\boldsymbol{w}_j)$. We call the nested ordered set $(T_{ij})_{i,j\in\mathscr{I}}$ the component form of $\boldsymbol{T}$ w.r.t. $(\boldsymbol{u}_i\otimes\boldsymbol{w}_j)_{i,j\in\mathscr{I}}$, and denote it as T.

From here on, unless otherwise specified, we will be following the Einstein summation convention. As per this convention a repeated index in a term will imply a sum over that term with the repeated index taking values in $\mathscr{I}$. For example, the expression $X_i\boldsymbol{E}_i$ represents the sum $\sum_{i\in\mathscr{I}} X_i\boldsymbol{E}_i$. And an unrepeated index in a term will signify a set of 3 terms. For example, the term $\boldsymbol{E}_i$ represents the set $\{\boldsymbol{E}_i \,|\, i \in \mathscr{I}\}$.

The operator $D_j\left[\cdot\right]$ is defined such that

$$\left\{D_j\left[f\right]\right\}\left[\mathbf{X}\right] = \frac{\partial f\left[\mathbf{X}\right]}{\partial X_j}, \tag{3.6}$$

for $f : \mathbb{R}^3 \to \mathbb{R}$. We abbreviate $D_j\left[f\right]$ as $D_j f$.

4 Mechanics Model

4.1 Kinematics

4.1.1 Reference configuration

We show our assumed geometries for the spheroid and agarose microwell region, which is close to the spheroid, in the reference (undeformed) configuration in Fig. 1 (a). As can be seen in Fig. 1 (a), the microwell's bottom has a hemispherical shape of radius R_1 m, and its inner sides have a cylindrical shape of radius R_1 m as well. The cortical spheroid, of course, has a spherical shape of radius of R_0 m. We assume that in the reference configuration the spheroid's center O_s lies on the well's central axis ℓ_c, and that the spheroid rests on the bottom of the well, with a single point touching (see Fig. 1 (a)). We take the microwell's central axis ℓ_c to be parallel to $\boldsymbol{E}_2$. As per the motion we assume for the continua, given by (2.1), in the reference configuration the central axis ℓ_c is perpendicular to the rotation axis $\boldsymbol{E}_3$[1].

Let

$$\kappa_{\mathrm{R}}\left[\mathscr{B}^{\mathrm{sprd}}\right] := \left\{\mathbf{X} \in \mathbb{R}^3 \mid X_i\boldsymbol{E}_i \in \boldsymbol{\kappa}_{\mathrm{R}}\left[\mathscr{B}^{\mathrm{sprd}}\right]\right\}, \tag{4.1}$$

and

$$\kappa_{\mathrm{R}}\left[\mathscr{B}^{\mathrm{gel}}\right] := \left\{\mathbf{X} \in \mathbb{R}^3 \mid X_i\boldsymbol{E}_i \in \boldsymbol{\kappa}_{\mathrm{R}}\left[\mathscr{B}^{\mathrm{gel}}\right]\right\}. \tag{4.2}$$

In our model, we take $R_1 = 2 \times 10^{-4}$, and $R_0 = 8 \times 10^{-5}$.

4.1.2 Deformation mapping

The motion of the continua is given by (2.1). To partially reiterate, the vector $\boldsymbol{X} \in \mathbb{E}_{\mathrm{R}}$ is the reference position vector of the material particle $\mathscr{X}$. The symbol $\boldsymbol{I}_{\mathbb{E}_{\mathrm{R}}\to\mathbb{E}}$ denote the identity linear map from $\mathbb{E}_{\mathrm{R}}$ onto $\mathbb{E}$. More explicitly, $\boldsymbol{I}_{\mathbb{E}_{\mathrm{R}}\to\mathbb{E}} = \boldsymbol{e}_i \otimes \boldsymbol{E}_i$. The symbol $\boldsymbol{Q}_{\boldsymbol{\tau}}$ denotes $Q_{ij}\left[\tau\right]\boldsymbol{e}_i \otimes \boldsymbol{e}_j$, where $Q_{ij}\left[\tau\right]$ are defined in (3.1). The map $\boldsymbol{U}^{\star} : \mathbb{E}_{\mathrm{R}} \to \mathbb{E}_{\mathrm{R}}$ is the intermediate displacement field of $\mathscr{B}$. The symbol $\boldsymbol{x}_{\boldsymbol{\tau}}\left[\boldsymbol{X}\right]$ is the material particle $\boldsymbol{X}$'s position vector in $\mathbb{E}$ at the time instance $\boldsymbol{\tau}$. The set $\boldsymbol{\kappa}_{\boldsymbol{\tau}}\left[\mathscr{B}\right] = \left\{\boldsymbol{x}_{\boldsymbol{\tau}}\left[\boldsymbol{X}\right] \in \mathbb{E} \,\middle|\, \boldsymbol{X} \in \boldsymbol{\kappa}_{\mathrm{R}}\left[\mathscr{B}\right]\right\}$ is called the current body.

As per (2.1) the continua's deformations are time invariant in the co-rotational basis $\left(\boldsymbol{e}_i\left[\tau\right]\right)_{i\in\mathscr{I}}$. The co-rotational basis themselves rotate about the time stationary vector $\boldsymbol{e}_3$ with the constant angular velocity $\omega_{\max}$ $\mathrm{rad/s}$ (see Fig. 1 (c)). (To recall, $\omega_{\max}$ $\mathrm{rad/s}$ is the angular velocity of the centrifuge in the second stage of the loading. In the experiments $\omega_{\max}$ is either 0 (control), 209, or 419.) Thus, as per our model the continua rotate about the $\boldsymbol{e}_3$ axis, at the constant angular velocity of $\omega_{\max}$, with a time invariant deformation.

[1] Note that in our problem the reference configuration and the configuration when the centrifuge is not spinning are not isomorphic. When the centrifuge is not spinning the central axis and the rotation axis are parallel to each other, where as in the reference configuration the central axis ℓ_c and the rotation axis $\boldsymbol{E}_3$ are perpendicular to each other.

4.1.3 Displacements components

Expressing $\boldsymbol{X} = X_i \boldsymbol{E}_i$, and $\boldsymbol{U}^\star[\boldsymbol{X}] = U_i^\star[\mathbf{X}]\,\boldsymbol{E}_i$, and using (2.1) and (3.3) it can be shown that

$$\boldsymbol{x}_\tau[\boldsymbol{X}] = \overline{x}_i[\mathbf{X}]\,\boldsymbol{e}_i[\tau], \tag{4.3a}$$

where

$$\overline{x}_i[\mathbf{X}] = X_i + U_i^\star[\mathbf{X}]. \tag{4.3b}$$

Denoting $(\overline{x}_i[\mathbf{X}])_{i\in\mathscr{I}}$ as $\overline{\mathbf{x}}[\mathbf{X}]$ and $(U_i^\star[\mathbf{X}])_{i\in\mathscr{I}}$ as $\mathbf{U}^\star[\mathbf{X}]$, (4.3b) can equivalently be expressed as

$$\overline{\mathbf{x}}[\mathbf{X}] = \mathbf{X} + \mathbf{U}^\star[\mathbf{X}]. \tag{4.4}$$

We refer to the restriction of $\overline{\mathbf{x}}[\cdot]$ to $\kappa_{\mathrm{R}}\left[\mathscr{B}^{\mathrm{sprd}}\right]$ as $\overline{\mathbf{x}}^{\mathrm{sprd}}[\cdot]$. The maps $\overline{\mathbf{x}}^{\mathrm{gel}}[\cdot]$ and $\overline{\mathbf{x}}^{\mathrm{fluid}}[\cdot]$ are defined similarly.

4.1.4 Velocity components

We call $\mathscr{L}(\mathbb{T},\mathbb{E})$ the physical velocity vector space and denote it as $\mathbb{V}$. It can be shown that the set $(\boldsymbol{v}_i[\tau])_{i\in\mathscr{I}}$, where $\boldsymbol{v}_i[\tau]\in\mathbb{V}$ and are defined such that $\{\boldsymbol{v}_i[\tau]\}\boldsymbol{\tau} = \tau\boldsymbol{e}_i[\tau]$, that is $\boldsymbol{v}_i[\tau] := \boldsymbol{e}_i[\tau]\otimes\boldsymbol{s}^*$, where $\boldsymbol{s}^*$ is the dual of $\boldsymbol{s}$, provides an orthonormal basis for $\mathbb{V}$. The velocity of a material particle $\boldsymbol{X}$ executing its motion in $\mathbb{E}$ lies in $\mathbb{V}$. The velocity of the material particle $\boldsymbol{X}$ at the instant $\boldsymbol{\tau}$, which we denote as $\boldsymbol{V}_{\boldsymbol{\tau}}[\boldsymbol{X}]$, equals the value of the Fréchet derivative of the map $\mathbb{T}\ni\boldsymbol{\tau}\mapsto\boldsymbol{x}_{\boldsymbol{X}}[\boldsymbol{\tau}]\in\mathbb{E}$, where $\boldsymbol{x}_{\boldsymbol{X}}[\boldsymbol{\tau}] = \boldsymbol{x}_{\boldsymbol{\tau}}[\boldsymbol{X}]$, at the time instance $\boldsymbol{\tau}$. Thus, it follows from (2.1) that for $\tau \geqslant 0$

$$\boldsymbol{V}_{\boldsymbol{\tau}}[\boldsymbol{X}] = V_i[\mathbf{X}]\,\boldsymbol{v}_i[\tau], \tag{4.5a}$$

where

$$V_i[\mathbf{X}] = W_{ij}\left(X_j + U_j^\star[\mathbf{X}]\right), \tag{4.5b}$$

and

$$W_{ij} = Q'_{kj}[\tau]Q_{ki}[\tau]. \tag{4.5c}$$

From (3.1) and (4.5c) it follows that

$$(W_{ij})_{i,j\in\mathscr{I}} = \begin{pmatrix} 0 & -\omega_{\max} & 0 \\ \omega_{\max} & 0 & 0 \\ 0 & 0 & 0 \end{pmatrix} =: \mathbf{W}. \tag{4.6}$$

Denoting $(V_i[\mathbf{X}])_{i\in\mathscr{I}}$ as $\mathbf{V}[\mathbf{X}]$, (4.5b) can equivalently be written as

$$\mathbf{V}[\mathbf{X}] = \mathbf{W}(\mathbf{X} + \mathbf{U}^\star[\mathbf{X}]). \tag{4.7}$$

The velocity of the material particle located at $\boldsymbol{x}\in\mathbb{E}$ at the time instance $\boldsymbol{\tau}$ is defined as

$$\boldsymbol{v}_{\boldsymbol{\tau}}[\boldsymbol{x}] = \boldsymbol{V}_\tau\left[\boldsymbol{x}_{\boldsymbol{\tau}}^{-1}[\boldsymbol{x}]\right]. \tag{4.8}$$

From $(4.5a)$, $(4.5b)$, and $(4.3b)$ the equation 4.8 can be written as

$$\boldsymbol{v}_{\boldsymbol{\tau}}[\boldsymbol{x}] = W_{ij}\breve{x}_j[\boldsymbol{x},\tau]\,\boldsymbol{v}_i[\tau]\,. \tag{4.9}$$

4.1.5 Accelerations

We call $\mathscr{L}(\mathbb{T},\mathbb{V})$ the physical acceleration vector space and denote it as $\mathbb{A}$. It can be shown that the set $(\boldsymbol{a}_i[\tau])_{i\in\mathscr{I}}$, where $\boldsymbol{a}_i[\tau]\in\mathbb{A}$ and are defined such that $\{\boldsymbol{a}_i[\tau]\}\boldsymbol{\tau} = \tau\boldsymbol{v}_i[\tau]$, i.e., $\boldsymbol{a}_i[\tau] = \boldsymbol{v}_i[\tau]\otimes\boldsymbol{s}^*$, provides an orthonormal basis for $\mathbb{A}$. The acceleration of a material particle $\boldsymbol{X}$ executing its motion in $\mathbb{E}$ lies in $\mathbb{A}$. The acceleration of $\boldsymbol{X}$ at the time instance $\boldsymbol{\tau}$ equals the value of the Fréchet derivative of the map $\mathbb{T}\ni\boldsymbol{\tau}\mapsto\boldsymbol{V}_{\boldsymbol{X}}(\boldsymbol{\tau})\in\mathbb{V}$, where $\boldsymbol{V}_{\boldsymbol{X}}(\boldsymbol{\tau}) = \boldsymbol{V}_{\boldsymbol{\tau}}(\boldsymbol{X})$, at the time instance $\boldsymbol{\tau}$. Thus, it follows from $(4.5a)$ that for $\tau \geqslant 0$

$$\boldsymbol{A}_\tau[\boldsymbol{X}] = A_i[\mathbf{X}]\,\boldsymbol{a}_i[\tau]\,, \tag{4.10a}$$

where

$$A_i[\mathbf{X}] = W_{im}W_{mp}\left(X_p + U_p^\star[\mathbf{X}]\right). \tag{4.10b}$$

Denoting $(A_i[\mathbf{X}])_{i\in\mathscr{I}}$ as $\mathbf{A}[\mathbf{X}]$, $(4.10b)$ can be equivalently be written as

$$\mathbf{A}[\mathbf{X}] = \mathbf{W}^2(\mathbf{X}+\mathbf{U}^\star[\mathbf{X}])\,. \tag{4.11}$$

The acceleration of the material particle located at $\boldsymbol{x}\in\mathbb{E}$ at the time instance $\boldsymbol{\tau}$ is defined as

$$\boldsymbol{a}_{\boldsymbol{\tau}}[\boldsymbol{x}] = \boldsymbol{A}_\tau\left[\boldsymbol{x}_{\boldsymbol{\tau}}^{-1}[\boldsymbol{x}]\right]. \tag{4.12}$$

From $(4.10a)$, $(4.10b)$, and $(4.3b)$ the equation 4.12 can be written as

$$\boldsymbol{a}_{\boldsymbol{\tau}}[\boldsymbol{x}] = W_{im}W_{mp}\breve{x}_p[\boldsymbol{x},\tau]\,\boldsymbol{a}_i[\tau]\,. \tag{4.13}$$

4.1.6 Deformation gradient and Strains

The deformation gradient corresponding to the deformation mapping $\boldsymbol{x}_{\boldsymbol{\tau}}[\cdot]$, given in (2.1), is

$$\{\boldsymbol{\nabla}_{\boldsymbol{X}}[\boldsymbol{x}_{\boldsymbol{\tau}}]\}[\boldsymbol{X}] =: \boldsymbol{F}_{\boldsymbol{\tau}}[\boldsymbol{X}] = F_{ij}[\mathbf{X}]\,\boldsymbol{e}_i[\tau]\otimes\boldsymbol{E}_j, \tag{4.14a}$$

where

$$F_{ij}[\mathbf{X}] := \delta_{ij} + D_jU_i^\star[\mathbf{X}]\,. \tag{4.14b}$$

The right Cauchy-Green deformation tensor corresponding to the deformation gradient $F_{ij}[\mathbf{X}]\,\boldsymbol{e}_i[\tau]\otimes\boldsymbol{E}_j$ is

$$\boldsymbol{C}[\boldsymbol{X}] = C_{ij}[\mathbf{X}]\,\boldsymbol{E}_i\otimes\boldsymbol{E}_j, \tag{4.15a}$$

where

$$C_{ij}[\mathbf{X}] = F_{mi}[\mathbf{X}]\,F_{mj}[\mathbf{X}]\,. \tag{4.15b}$$

We abbreviate $(F_{ij}[\mathbf{X}])_{i,j\in\mathscr{I}}$ and $(C_{ij}[\mathbf{X}])_{i,j\in\mathscr{I}}$ as $\mathbf{F}[\mathbf{X}]$ and $\mathbf{C}[\mathbf{X}]$, respectively.

4.2 Equilibrium

4.2.1 Cauchy-momentum equations in the reference body

It follows from the principle of balance of linear momentum and our modeling decision *d.5* that

$$\{\mathrm{Div}\, \boldsymbol{F}_{\boldsymbol{\tau}} \boldsymbol{S}\}\, [\boldsymbol{X}] = \rho_{\mathrm{o}} \boldsymbol{A}_{\tau}\, [\boldsymbol{X}]\,, \tag{4.16}$$

where $\{\mathrm{Div}\, \boldsymbol{F}_{\boldsymbol{\tau}} \boldsymbol{S}\}\, [\cdot]$ is the divergence of the field $\boldsymbol{X} \mapsto \boldsymbol{F}_{\boldsymbol{\tau}}\, [\boldsymbol{X}]\, \boldsymbol{S}\, [\boldsymbol{X}]$. Here $\boldsymbol{S}\, [\boldsymbol{X}]$ is the 2nd Piola-Kirchhoff stress tensor at the material particle $\boldsymbol{X}$.

In component form (4.16) can be written as

$$\{D_j\, [F_{im} S_{mj}]\}\, [\mathbf{X}] = \rho_0 A_i\, [\mathbf{X}]\,, \tag{4.17}$$

where $S_{ij}\, [\mathbf{X}]$, $i,j \in \mathscr{I}$, are the components of $\boldsymbol{S}\, [\boldsymbol{X}]$[2].

In (4.17) replacing $A_i\, [\mathbf{X}]$ with the RHS of $(4.10\mathrm{b})$ we get

$$\{D_j\, [F_{im} S_{mj}]\}\, [\mathbf{X}] = \rho_0 W_{im} W_{mp} \left(X_p + U_p^{\star}\, [\mathbf{X}]\right)\,, \tag{4.18}$$

where $\rho_0\ \mathrm{kg/m^3}$ is the density of the continua and $\rho_0 = 1240$ for the cortical spheroid; $\rho_0 = 1640$ for the agarose hydrogel; $\rho_0 = 980$ for the cortical media.

Noting from (4.6) that $W_{im} W_{mp} = -\omega_{\max}^2 \left(\delta_{ip} - \delta_{i3} \delta_{3p}\right)$ in (4.18) we get

$$\{D_j\, [F_{im} S_{mj}]\}\, [\mathbf{X}] = -\rho_0 \omega_{\max}^2 \left(X_i + U_i^{\star}\, [\mathbf{X}] - \delta_{i3} \left(X_3 + U_3^{\star}\, [\mathbf{X}]\right)\right). \tag{4.19}$$

The domain of (4.19) is either $\kappa_{\mathrm{R}} \left[\mathscr{B}^{\mathrm{sprd}}\right]$, or $\kappa_{\mathrm{R}} \left[\mathscr{B}^{\mathrm{gel}}\right]$, which were, respectively, defined in (4.1) and (4.2). Irrespective, of whether $\mathbf{X}$ belongs to $\kappa_{\mathrm{R}} \left[\mathscr{B}^{\mathrm{sprd}}\right]$ or $\kappa_{\mathrm{R}} \left[\mathscr{B}^{\mathrm{gel}}\right]$ the co-ordinates X_1 and X_3 are always less than $L_3/2$, where, as we have described in the main manuscript, L_3 is the distance between the central axes of adjacent microwells, and is 800 microns in our model. The co-ordinate X_2 in the domains, however, varies between 176 and 179 millimeters. (Figures 1 and 2 in the main manuscript can help in understanding how we arrived at these ranges for the different co-ordinates.) Therefore, in (4.19) we ignore X_1, and X_3, in comparison to X_2. **(a.6)** The intermediate displacement components $U_i^{\star}\, [\mathbf{X}]$ are unlikely to be larger than the height of the hydrogel, which is, roughly, 3 mm. Therefore, we also ignore $U_i^{*}\, [\mathbf{X}]$ in comparison to X_2 in (4.19). In summary, we approximate (4.19) as

$$\{D_j\, [F_{im} S_{mj}]\}\, [\mathbf{X}] = -\rho_0 \omega_{\max}^2 \delta_{i2} \delta_{2j} X_j. \tag{4.20}$$

4.2.2 Cauchy-momentum equations in the current body

It follows from the principle of balance of linear momentum that

$$\{\mathrm{Div}\, \boldsymbol{T}_{\boldsymbol{\tau}}\}\, [\boldsymbol{x}] = \rho_{\tau}\, [\boldsymbol{x}]\, \boldsymbol{a}_{\boldsymbol{\tau}}\, [\boldsymbol{x}]\,, \tag{4.21}$$

for all $\boldsymbol{x} \in \boldsymbol{\kappa}_{\boldsymbol{\tau}}\, [\mathscr{B}]$, where $\{\mathrm{Div}\, \boldsymbol{T}_{\boldsymbol{\tau}}\}\, [\cdot]$ is the divergence of the field $\boldsymbol{x} \mapsto \boldsymbol{T}_{\boldsymbol{\tau}}\, [\boldsymbol{x}]$. Here $\boldsymbol{T}_{\boldsymbol{\tau}}\, [\boldsymbol{x}]$ is the Cauchy stress tensor at the current position $\boldsymbol{x}$ at the time instance $\boldsymbol{\tau}$. And $\rho_{\tau}\, [\boldsymbol{x}] := \rho_0 / \mathrm{Det} \left[\boldsymbol{F}_{\boldsymbol{\tau}} \left[\boldsymbol{x}_{\boldsymbol{\tau}}^{-1}\, [\boldsymbol{x}]\right]\right]$, where $\mathrm{Det}\, [\cdot]$ is the determinant operator.

In (4.21) replacing $\boldsymbol{a}_{\boldsymbol{\tau}}\, [\boldsymbol{x}]$ with the RHS of (4.13) and rewriting (4.21) in component form, we get

$$\{D_j T_{ij}\}\, [\mathbf{x}] = \overline{\rho}_{\tau}\, [\mathbf{x}]\, W_{im} W_{mp} x_p, \tag{4.22}$$

[2] Here we omit providing the mathematical details of how precisely $S_{ij}\, [\mathbf{X}]$ and $T_{ij}\, [\mathbf{x}]$ are, respectively, related to $\boldsymbol{S}\, [\boldsymbol{X}]$ and $\boldsymbol{T}_{\boldsymbol{\tau}}\, [\boldsymbol{x}]$. Since doing so will require notions from *exterior algebra* and *differential geometry* that need a significant amount of space to properly explain, and hence would distract from the primary focus of this paper.

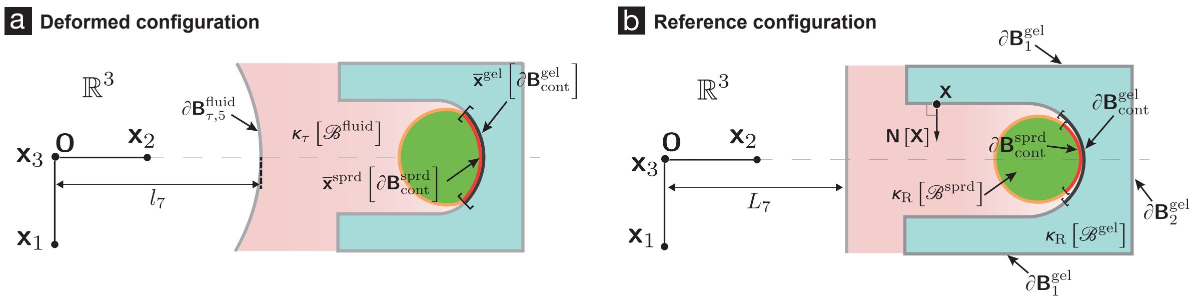

Figure 2 : An illustration of the cortical spheroid (green), cortical fluid media (pink), and the agarose hydrogel microwell surfaces (cyan) in the deformed (a) and reference (b) configurations.

for all $\mathbf{x} \in \kappa_\tau[\mathscr{B}]$,

$$\kappa_\tau[\mathscr{B}] := \left\{\mathbf{x} := (x_1, x_2, x_3) \in \mathbb{R}^3 \mid x_i \boldsymbol{e}_i[\tau] \in \boldsymbol{\kappa_\tau}[\mathscr{B}]\right\}, \tag{4.23}$$

and $T_{ij}[\mathbf{x}]$, $i,j \in \mathscr{I}$, are the components of $\boldsymbol{T_\tau}[x]^2$. Here $\overline{\rho}_\tau[\cdot]$ is defined such that $\overline{\rho}_\tau[\mathbf{x}] = \rho_\tau[x_i \boldsymbol{e}_i[\tau]]$. Noting from (4.6) that $W_{im}W_{mp} = -\omega_{\max}^2(\delta_{ip} - \delta_{i3}\delta_{3p})$ in (4.22) we get

$$\{D_j T_{ij}\}[\mathbf{x}] = -\overline{\rho}_\tau[\mathbf{x}]\,\omega_{\max}^2(x_i - \delta_{i3}x_3). \tag{4.24}$$

4.3 Pressure in the cortical media

We model the cortical media as an incompressible Newtonian fluid. It can be shown that in our problem, the rate of deformation tensor is naught (see §A for details). Consequently, from [1, §22], we can get

$$\mathbf{T}[\mathbf{x}] = -p_s^{\text{fluid}}[\mathbf{x}]\,\mathbf{I}_{3\times 3}, \tag{4.25}$$

for all $\mathbf{x} \in \kappa_\tau\left[\mathscr{B}^{\text{fluid}}\right]$,

$$\kappa_\tau\left[\mathscr{B}^{\text{fluid}}\right] := \left\{\mathbf{x} \in \mathbb{R}^3 \mid x_i \boldsymbol{e}_i[\tau] \in \boldsymbol{\kappa_\tau}\left[\mathscr{B}^{\text{fluid}}\right]\right\}, \tag{4.26}$$

and $\mathbf{T}[\mathbf{x}] := (T_{ij}[\mathbf{x}])_{i,j\in\mathscr{I}}$ and $p_s^{\text{fluid}}[\cdot]$ is the pressure field.

In (4.24) substituting $\overline{\rho}_\tau[\mathbf{x}]$ as ρ_0, since we have assumed the cortical media as being incompressible, and $\mathbf{T}[\mathbf{x}]$ as $-p_s^{\text{fluid}}[\mathbf{x}]\,\mathbf{I}_{3\times 3}$ from (4.25), we get that

$$\left\{D_i\left[p_s^{\text{fluid}}\right]\right\}[\mathbf{x}] = \rho_0\omega_{\max}^2(x_i - \delta_{i3}x_3), \quad \forall \mathbf{x} \in \kappa_\tau\left[\mathscr{B}^{\text{fluid}}\right]. \tag{4.27}$$

It can be shown that in our problem the free surface of the fluid at the time instance $\boldsymbol{\tau}$ (marked as $\partial\mathbf{B}_{\tau,5}^{\text{fluid}}$ in Fig. 2 (a)) is always part of a cylinder. More specifically, it can be shown that

$$\partial\mathbf{B}_{\tau,5}^{\text{fluid}} = \{\mathbf{x} \in \kappa_\tau\left[\mathscr{B}^{\text{fluid}}\right] \mid x_1^2 + x_2^2 = l_7^2\}. \tag{4.28}$$

The parameter l_7 in 4.28 is the distance of the center of $\partial\mathbf{B}_{\tau,5}^{\text{fluid}}$ from the rotation axis (see Fig. 2 (a)).

The surface $\partial\mathbf{B}_{\tau,5}^{\text{fluid}}$ experiences the atmospheric pressure p^{atm} Pa, where $p^{\text{atm}} = 1.01325 \times 10^5$. Hence, one of the boundary conditions on $p_s^{\text{fluid}}[\cdot]$ is

$$p_s^{\text{fluid}}[\mathbf{x}] = p^{\text{atm}}, \quad \mathbf{x} \in \partial\mathbf{B}_{\tau,5}^{\text{fluid}}. \tag{4.29}$$

Solving (4.27) with the boundary condition (4.29) we get that

$$p_s^{\text{fluid}}[\mathbf{x}] = \frac{1}{2}\rho_0\omega_{\max}^2\left(x_1^2 + x_2^2 - l_7^2\right) + p^{\text{atm}}. \tag{4.30}$$

4.4 Material constitutive laws

4.4.1 Constitutive law for the spheroid

In (4.20) when $\mathbf{X} \in \kappa_{\mathrm{R}}\left[\mathscr{B}^{\mathrm{sprd}}\right]$

$$S_{ij}\left[\mathbf{X}\right] = \breve{S}_{ij}^{\mathrm{sprd}}\left[\mathbf{C}\left[\mathbf{X}\right]\right] - p_m^{\mathrm{sprd}}\left[\mathbf{X}\right] J\left[\mathbf{C}\left[\mathbf{X}\right]\right]\left(\mathbf{C}\left[\mathbf{X}\right]\right)^{-1}, \tag{4.31a}$$

where $S_{ij}\left[\mathbf{X}\right]$ is the i-j^{th} component of $\boldsymbol{S}\left[\boldsymbol{X}\right]$, the 2nd Piola Kirchhoff stress at the material particle $\boldsymbol{X}$,

$$J\left[\mathbf{C}\right] := \sqrt{\mathsf{Det}\left[\mathbf{C}\right]}, \tag{4.31b}$$

$\breve{S}_{ij}^{\mathrm{sprd}}\left[\cdot\right]$ is the i-j^{th} component of $\breve{\mathbf{S}}^{\mathrm{sprd}}\left[\cdot\right]$,

$$\breve{\mathbf{S}}^{\mathrm{sprd}}[\mathbf{C}] = \mu\, \mathsf{Det}\left[\mathbf{C}\right]^{-\frac{1}{3}}\left(\mathbf{I}_{3\times 3} - \frac{1}{3}\,\mathsf{Tr}\left[\mathbf{C}\right]\mathbf{C}^{-1}\right), \tag{4.31c}$$

and μ Pa is the shear modulus. In our model, we take $\mu = 1.33 \times 10^3$ for the cortical spheroid [5]. Here $\mathsf{Tr}\left[\cdot\right]$ is the trace operator. In $(4.31\mathrm{a})$ the quantity $p_m^{\mathrm{sprd}}\left[\mathbf{X}\right]$ acts as a Lagrange undetermined multiplier, which can be interpreted as the hydrostatic pressure at the material particle $\mathbf{X}$. Since $p_m^{\mathrm{sprd}}\left[\cdot\right]$ is an unknown *a priori*, we solve (4.20) in conjunction with the incompressibility constraint

$$J\left[\mathbf{C}\left[\mathbf{X}\right]\right] = 1. \tag{4.32}$$

Equations (4.32) are the incompressible neo-Hookean material model from [6, equation 5.50].

4.4.2 Constitutive law for the agarose hydrogel

In (4.20) when $\mathbf{X} \in \kappa_{\mathrm{R}}\left[\mathscr{B}^{\mathrm{gel}}\right]$

$$S_{ij}[\mathbf{X}] = \breve{S}_{ij}^{\mathrm{gel}}\left[\mathbf{C}\left[\mathbf{X}\right]\right], \tag{4.33a}$$

where $\breve{S}_{ij}^{\mathrm{gel}}\left[\cdot\right]$ is the i-j^{th} component of $\breve{\mathbf{S}}^{\mathrm{gel}}\left[\cdot\right]$,

$$\breve{\mathbf{S}}^{\mathrm{gel}}\left[\mathbf{C}\right] = \mu\, \mathsf{Det}\left[\mathbf{C}\right]^{-1/3}\left(\mathbf{I}_{3\times 3} - \frac{1}{3}\,\mathsf{Tr}\left[\mathbf{C}\right]\mathbf{C}^{-1}\right) + \left(\lambda + \frac{2}{3}\mu\right) J\left[\mathbf{C}\right]\left(J\left[\mathbf{C}\right] - 1\right)\mathbf{C}^{-1}. \tag{4.33b}$$

Here λ Pa and μ Pa are the Lame's parameters and we take $\lambda = 4.28571 \times 10^5$ and $\mu = 1.07143 \times 10^5$ from [7, 8]. Equations (4.33) are the compressible neo-Hookean material model from [9, §3.5.5].

4.5 Boundary conditions

The solution of (4.20) also requires the use of the following boundary conditions.

Let $\partial\mathbf{B}^{\mathrm{sprd}}$ and $\partial\mathbf{B}^{\mathrm{gel}}$ be the surfaces of the spheroid $\kappa_{\mathrm{R}}\left[\mathscr{B}^{\mathrm{sprd}}\right]$ and the agarose hydrogel $\kappa_{\mathrm{R}}\left[\mathscr{B}^{\mathrm{gel}}\right]$, respectively. Let $\partial\mathbf{B}_{\mathrm{cont}}^{\mathrm{sprd}}$ and $\partial\mathbf{B}_{\mathrm{cont}}^{\mathrm{gel}}$ be the surfaces of $\kappa_{\mathrm{R}}\left[\mathscr{B}^{\mathrm{sprd}}\right]$ and $\kappa_{\mathrm{R}}\left[\mathscr{B}^{\mathrm{gel}}\right]$, respectively, that come into contact with each other (see Fig. 2 (b)). They are both unknown *a priori*. The boundary conditions on $\partial\mathbf{B}_{\mathrm{cont}}^{\mathrm{sprd}}$ and $\partial\mathbf{B}_{\mathrm{cont}}^{\mathrm{gel}}$ are that there are no shear tractions on them, and the displacements of the spheroid and gel on them respectively are such that

$$\overline{\mathbf{x}}^{\mathrm{sprd}}\left[\partial\mathbf{B}_{\mathrm{cont}}^{\mathrm{sprd}}\right] = \overline{\mathbf{x}}^{\mathrm{gel}}\left[\partial\mathbf{B}_{\mathrm{cont}}^{\mathrm{gel}}\right], \tag{4.34}$$

see Fig. 2 (a) for an illustration.

Let $\partial\mathbf{B}_1^{\mathrm{gel}}$ and $\partial\mathbf{B}_2^{\mathrm{gel}}$ be the surfaces of $\kappa_{\mathrm{R}}\left[\mathscr{B}^{\mathrm{gel}}\right]$ shown in Fig. 2 (b). The boundary conditions on $\partial\mathbf{B}^{\mathrm{sprd}}\backslash\partial\mathbf{B}_{\mathrm{cont}}^{\mathrm{sprd}}$ and $\partial\mathbf{B}^{\mathrm{gel}}\backslash\partial\mathbf{B}_{\mathrm{cont}}^{\mathrm{gel}}\backslash\bigcup_{i=1}^{2}\partial\mathbf{B}_i^{\mathrm{gel}}$ (see Fig. 2 (b)), due to the spheroid's and gel's, respective,

interactions with the fluid media are

$$\left(\mathbf{C}[\mathbf{X}]\,\mathbf{S}[\mathbf{X}] - p_m^{\text{fluid}}[\mathbf{X}]\,\mathbf{I}_{3\times 3}\right)\mathbf{N}[\mathbf{X}] = \mathbf{0}_{3\times 1}, \tag{4.35a}$$

where $\mathbf{S}[\mathbf{X}] := (S_{ij}[\mathbf{X}])_{i,j\in\mathscr{I}}$, $\mathbf{0}_{3\times 1} \equiv (0,0,0)$, and $\mathbf{N}[\mathbf{X}]$ is the unit outward surface normal vector at the location $\mathbf{X}$ (see Fig. 2 (b) for example). The field $p_m^{\text{fluid}}[\mathbf{X}]$ in (4.35a) is $p_s^{\text{fluid}}[\overline{\mathbf{x}}[\mathbf{X}]]$, where $p_s^{\text{fluid}}[\cdot]$ is given in (4.30). More concretely,

$$p_m^{\text{fluid}}[\mathbf{X}] = \frac{1}{2}\rho_0\omega_{\max}^2\left((X_1 + U_1^\star[\mathbf{X}])^2 + (X_2 + U_2^\star[\mathbf{X}])^2 - l_7^2\right) + p^{\text{atm}}. \tag{4.35b}$$

We cannot independently calculate l_7 in our model. Therefore, in (4.35b) we approximate l_7 as L_7, the distance of the fluid's free surface under the assumption that none of the continua (the spheroid, the fluid, and the agarose gel) deform (see Fig. 2 (b)). Also, since $U_1^\star$, X_1, and $U_2^\star$ are much smaller than X_2, in (4.35b) we approximate $(X_1 + U_1^\star[\mathbf{X}])$ as naught, and $(X_2 + U_2^\star[\mathbf{X}])$ as X_2. In summary, we compute

$$p_m^{\text{fluid}}[\mathbf{X}] \approx \frac{1}{2}\rho_0\omega_{\max}^2\left(X_2^2 - L_7^2\right) + p^{\text{atm}}. \tag{4.35c}$$

An additional boundary condition on the agarose gel is that

$$U_1^\star[\mathbf{X}]\,X_1 = -U_3^\star[\mathbf{X}]\,X_3, \quad \forall \mathbf{X} \in \partial\mathbf{B}_1^{\text{gel}} \tag{4.36a}$$

$$U_2^\star[\mathbf{X}] = 0, \quad \forall \mathbf{X} \in \partial\mathbf{B}_2^{\text{gel}}. \tag{4.36b}$$

The boundary condition (4.36a) is a consequence of setting the radial component of the displacement field on $\partial\mathbf{B}_1^{\text{gel}}$ (see Fig. 2 (b)) to be naught, which we do to model the periodic arrangement of the agarose microwells and respect our assumption of the deformations being axi-symmetric in each hydrogel cell (see Fig. 1 in main manuscript). We choose the boundary condition (4.36b) to model the fact that the microwell sits on a polystyrene plate, which constrains its deformation in the $\boldsymbol{E}_2$ direction on $\partial\mathbf{B}_2^{\text{gel}}$.

5 Coupled boundary value problems

As per our model, the motion of the spheroid and the agarose hydrogel is given by the family of deformation maps $\boldsymbol{x}_\tau[\cdot]$. This family of deformation maps can be constructed using (4.3) once the displacement field components $U_i^\star[\cdot]$ are known. The restrictions of $U_i^\star[\cdot]$ to $\kappa_{\text{R}}\left[\mathscr{B}^{\text{sprd}}\right]$ (resp. $\kappa_{\text{R}}\left[\mathscr{B}^{\text{gel}}\right]$) are obtained by solving the partial differential equation (PDE) (4.20) over the region $\kappa_{\text{R}}\left[\mathscr{B}^{\text{sprd}}\right]$ (resp. $\kappa_{\text{R}}\left[\mathscr{B}^{\text{gel}}\right]$). We refer to the PDE (4.20) posed over the region $\kappa_{\text{R}}\left[\mathscr{B}^{\text{sprd}}\right]$ as the spheroid boundary value problem (BVP), and the PDE (4.20) posed over the region $\kappa_{\text{R}}\left[\mathscr{B}^{\text{gel}}\right]$ as the gel BVP. Recall that the functions $\{F_{im}S_{mj}\}[\cdot]$ appearing in (4.20) are defined as

$$\mathbf{X} \mapsto F_{im}[\mathbf{X}]\,S_{mj}[\mathbf{X}]. \tag{5.1}$$

In the spheroid (resp. gel) BVP the $F_{im}[\cdot]$ in (5.1) are to be interpreted as the restrictions of the $F_{im}[\cdot]$ defined in (4.14b) to $\kappa_{\text{R}}\left[\mathscr{B}^{\text{sprd}}\right]$ (resp. $\kappa_{\text{R}}\left[\mathscr{B}^{\text{gel}}\right]$). For the spheroid (resp. gel) BVP the $S_{ij}[\cdot]$ in (5.1) is given by the function (4.31) (resp. (4.33)). In the spheroid BVP, due to the presence of the Lagrange multiplier (pressure field) $p_m^{\text{sprd}}[\cdot]$ in (4.31) the PDE (4.20) needs to be solved jointly with the incompressibility constraint equation (4.32). The boundary conditions in the spheroid and the gel BVPs are detailed in §4.5.

Note that the contact boundary condition (4.34) is part of both the spheroid as well as the gel BVPs. It couples the two BVPs, since it involves displacement components from both BVPs. Therefore, the

two BVPs cannot be solved independently. We solve the spheroid and the gel BVPs simultaneously using finite element techniques.

A Vanishing of the rate of deformation tensor

The rate of deformation tensor is defined as

$$\boldsymbol{D}_{\boldsymbol{\tau}}\left[\boldsymbol{x}\right]=\frac{1}{2}\left(\boldsymbol{L}_{\boldsymbol{\tau}}\left[\boldsymbol{x}\right]+\boldsymbol{L}_{\boldsymbol{\tau}}\left[\boldsymbol{x}\right]^{\mathsf{T}}\right), \tag{A.1}$$

where $\boldsymbol{L}_{\boldsymbol{\tau}}\left[\boldsymbol{x}\right]$ is the spatial velocity gradient, defined by

$$\boldsymbol{L}_{\boldsymbol{\tau}}\left[\boldsymbol{x}\right]=\left\{\boldsymbol{\nabla}_{\boldsymbol{x}}\boldsymbol{v}_{\boldsymbol{\tau}}\right\}\left[\boldsymbol{x}\right]. \tag{A.2}$$

From (4.9), $(\mathrm{A}.2)$ can be written as

$$\boldsymbol{L}_{\boldsymbol{\tau}}\left[\boldsymbol{x}\right]=W_{ij}\left[\tau\right]\boldsymbol{v}_{i}\left[\tau\right]\otimes\boldsymbol{e}_{j}\left[\tau\right]. \tag{A.3}$$

From the definition of W_{ij} (4.6), it follows that $W_{ij}=-W_{ji}$. Then from $(\mathrm{A}.3)$ and $(\mathrm{A}.1)$ we get that

$$\boldsymbol{D}_{\boldsymbol{\tau}}\left[\boldsymbol{x}\right]=\mathbf{0}. \tag{A.4}$$

References

[1] M. E. Gurtin, An introduction to continuum mechanics, Academic press, 1982.

[2] Y. Wan, W. Fang, R. W. Carlsen, H. Kesari, A finite rotation, small strain 2D elastic head model, with applications in mild traumatic brain injury, Journal of the Mechanics and Physics of Solids 179 (2023) 105362.

[3] Y. Wan, A. L. Fawzi, H. Kesari, Determining rigid body motion from accelerometer data through the square-root of a negative semi-definite tensor, with applications in mild traumatic brain injury, Computer Methods in Applied Mechanics and Engineering 390 (2022) 114271.

[4] M. M. Rahaman, W. Fang, A. L. Fawzi, Y. Wan, H. Kesari, An accelerometer-only algorithm for determining the acceleration field of a rigid body, with application in studying the mechanics of mild traumatic brain injury, Journal of the Mechanics and Physics of Solids 143 (2020) 104014.

[5] T. Boulet, M. L. Kelso, S. F. Othman, Microscopic magnetic resonance elastography of traumatic brain injury model, Journal of Neuroscience Methods 201 (2) (2011) 296–306.

[6] J. Bonet, R. D. Wood, Nonlinear continuum mechanics for finite element analysis, Cambridge university press, 1997.

[7] Y. Mori, S. Kanazawa, M. Watanabe, H. Suenaga, K. Okubo, S. Nagata, Y. Fujihara, T. Takato, K. Hoshi, Usefulness of agarose mold as a storage container for three-dimensional tissue-engineered cartilage, Materials Sciences and Applications 4 (2013) 73–78.

[8] V. Normand, D. L. Lootens, E. Amici, K. P. Plucknett, P. Aymard, New insight into agarose gel mechanical properties, Biomacromolecules 1 (4) (2000) 730–738.

[9] A. F. Bower, Applied mechanics of solids, CRC press, 2009.
